# N6-methyladenosine-driven miR-143/145-KLF4 circuit orchestrates the phenotypic switch of pulmonary artery smooth muscle cells

**DOI:** 10.1007/s00018-024-05304-1

**Published:** 2024-06-12

**Authors:** Kang Kang, Chuannan Sun, Hui Li, Xiaojia Liu, Jingyuan Deng, Silei Chen, Le Zeng, Jiahao Chen, Xinyi Liu, Jiahao Kuang, Jingjing Xiang, Jingqian Cheng, Xiaoyun Liao, Mujin Lin, Xingshi Zhang, Chuzhi Zhan, Sisi Liu, Jun Wang, Yanqin Niu, Cuilian Liu, Cai Liang, Jinsheng Zhu, Shuxin Liang, Haiyang Tang, Deming Gou

**Affiliations:** 1 Department of Biochemistry and Molecular Biology, Shenzhen University Medical School, Shenzhen, 518060 Guangdong China; 2 Shenzhen Key Laboratory of Microbial Genetic Engineering, Vascular Disease Research Center, College of Life Sciences and Oceanography, Guangdong Provincial Key Laboratory of Regional Immunity and Disease, Carson International Cancer Center, School of Medicine, Shenzhen University, Shenzhen, 518060 China; 3 State Key Laboratory of Respiratory Disease, National Clinical Research Center for Respiratory Disease, Guangzhou Institute of Respiratory Health, First Affiliated Hospital of Guangzhou Medical University, Guangzhou, 510120 Guangdong China

**Keywords:** Pulmonary arterial hypertension, microRNA, Non-coding RNA, Methylation, Transcription factor

## Abstract

Pulmonary hypertension (PH) is characterized by vascular remodeling predominantly driven by a phenotypic switching in pulmonary artery smooth muscle cells (PASMCs). However, the underlying mechanisms for this phenotypic alteration remain incompletely understood. Here, we identified that RNA methyltransferase METTL3 is significantly elevated in the lungs of hypoxic PH (HPH) mice and rats, as well as in the pulmonary arteries (PAs) of HPH rats. Targeted deletion of *Mettl3* in smooth muscle cells exacerbated hemodynamic consequences of hypoxia-induced PH and accelerated pulmonary vascular remodeling in vivo. Additionally, the absence of METTL3 markedly induced phenotypic switching in PASMCs in vitro. Mechanistically, METTL3 depletion attenuated m^6^A modification and hindered the processing of pri-miR-143/145, leading to a downregulation of miR-143-3p and miR-145-5p. Inhibition of hnRNPA2B1, an m^6^A mediator involved in miRNA maturation, similarly resulted in a significant reduction of miR-143-3p and miR-145-5p. We demonstrated that miR-145-5p targets Krüppel-like factor 4 (KLF4) and miR-143-3p targets fascin actin-bundling protein 1 (FSCN1) in PASMCs. The decrease of miR-145-5p subsequently induced an upregulation of KLF4, which in turn suppressed miR-143/145 transcription, establishing a positive feedback circuit between KLF4 and miR-143/145. This regulatory circuit facilitates the persistent suppression of contractile marker genes, thereby sustaining PASMC phenotypic switch. Collectively, hypoxia-induced upregulation of METTL3, along with m^6^A mediated regulation of miR-143/145, might serve as a protective mechanism against phenotypic switch of PASMCs. Our results highlight a potential therapeutic strategy targeting m^6^A modified miR-143/145-KLF4 loop in the treatment of PH.

**Supplementary Information:**

The online version contains supplementary material available at 10.1007/s00018-024-05304-1.

## Introduction

Pulmonary hypertension (PH) is a severe vascular lung disease characterized by vascular remodeling, predominantly attributed to abnormalities in pulmonary artery smooth muscle cells (PASMCs) [1]. Under typical physiological conditions, PASMCs exhibit a quiescent and differentiated phenotype, expressing contractile proteins such as α-SMA, SM22, Smoothelin and Calponin. However, in response to pathological stimuli like hypoxia and vascular injury, PASMCs undergo a phenotypic switching characterized by heightened proliferation, migration, and a decrease in contractile markers [2].

N6-methyladenosine (m^6^A) is a prevalent RNA modification implicated in various biological processes including mRNA splicing [3], stability [4], nuclear translocation [5], and translation initiation [6]. RNA m^6^A methylation encompasses a range of effector proteins: 'writers' like METTL3/14, Wtap, and KIAA1429; 'erasers' such as FTO and Alkbh5; and 'readers' including YTHDF1/2/3 and YTHDC1/2 [7]. RNA modification has been identified as a key player in the regulation of VSMCs-associated phenotypic alterations. For instance, METTL3 knockdown impedes the differentiation of adipose-derived stem cells (ADSCs) into VSMCs through regulating the secretion of specific paracrine factors [8]. Under the stimulation of indoxyl sulfate, METTL14 installs m^6^A on vascular osteogenic transcript Klotho, inducing its degradation and thereby promoting calcification of primary human artery smooth muscle cells (HASMCs) [9]. In PH samples and hypoxic PASMCs, elevated YTHDF1 accelerates PASMC proliferation, phenotype switch and PH progression by recognizing and boosting translational efficiency of m^6^A-modified MAGED1 [10]. Despite existing evidence on m^6^A involvement in VSMCs phenotype transformation, its role in mediating the phenotypic changes of PASMCs via microRNA remains inadequately explored.

microRNAs (miRNAs) are small, single-stranded, non-coding RNAs that negatively regulate gene expression [11]. Research highlights the pivotal role of miRNAs in the differentiation of vascular smooth muscle cells (VSMCs), especially miR-143/145 [12]. miR-143/145 are co-transcribed from a bicistronic transcript and are primarily expressed in VSMCs [13–15]. Their overexpression enhances the expression of VSMCs differentiation marker genes [15, 16]. Conversely, the absence of miR-143/145 results in a shift from a contractile to a synthetic phenotype in VSMCs [14, 17]. While several transcription factors such as SRF, Myocd, Nkx2-5 and MRTF-A regulate the expression of miR-143/145 [15, 17, 18], the effect of RNA methylation on these miRNAs during phenotypic changes remains largely unknown.

This study examines the role of METTL3-mediated m^6^A dynamic modification on miRNA-143/145 cluster relevant to phenotypic switching of PASMCs and pulmonary vascular remodeling. We demonstrated that METTL3 deficiency induces a pronounced phenotypic transition in PASMCs in vitro and promotes pulmonary vascular remodeling in vivo. The absence of METTL3 remarkably downregulates the expression of miR-143/145 cluster through regulating m^6^A-mediated miRNA processing. Furthermore, a positive regulatory circuit exists between KLF4 and miR-143/145. Thus, targeting the m^6^A-modification pathway associated with miR-143/145-KLF4 feedback loop presents a potential therapeutic strategy for PH treatment.

## Materials and methods

### Experimental animal model

Wild-type C57BL/6 mice and Sprague–Dawley (SD) rats were purchased from the Animal Center of Guangdong Province (China). Conditional C57BL/6 *Mettl3* knockout mice were generated by inserting loxP sites around exon 2/3 in *Mettl3* genomic DNA. The conditional *Mettl3* knockout mice *Mettl3*^*flox/flox*^ (*Mettl3*^*fl/fl*^) were crossed with tamoxifen-inducible *SMMHC* promoter-driven Cre line (*SMMHC-Cre*^*ERT2*^) to generate smooth muscle cell-specific *Mettl3* knockout mice (*SMMHC-Cre*^*ERT2*^*;Mettl3*^*fl/fl*^). *Mettl3*^*fl/fl*^ littermates served as controls. Tamoxifen was administrated vial intraperitoneal injection (20 mg/kg/day × 5 days, i.p.) one week before hypoxic treatment.

To generate hypoxia-induced PH model, 6-week-old male mice or SD rats were randomly assigned to groups and fed under normoxia (21% O_2_) or hypoxia (10% O_2_) condition for three weeks, respectively. After anesthetizing with 10% Chloral hydrate (0.3–0.4 mL/100 g), experimental animals were subsequently applied for hemodynamics and histological analysis.

### Plasmid construction and lentivirus (LV) production

Lentiviral shRNA and overexpression vectors were constructed using a modified Lenti-X vector (Clontech), employing the U6 promoter for shRNA expression and cytomegalovirus (CMV) promoter for controlling cDNA and the primary miRNAs (pri-miRNAs). The coding sequences (CDSs) of METTL3 (NM_019852.5), hnRNPA2B1 (NM_002137) and KLF4 (NM_053713.1) along with both wild-type and mutant human pri-miR-143 and pri-miR-145 sequences were inserted into the lentiviral vector to generate the respective overexpression vectors of pLV-CMV-METTL3, pLV-CMV-hnRNPA2B1, pLV-CMV-KLF4, pLV-CMV-pri-miR-143, pLV-CMV-pri-miR-143 mut, pLV-CMV-pri-miR-145, and pLV-CMV-pri-miR-145 mut. A lentiviral backbone lacking foreign genes downstream of the CMV promoter served as the negative control (OE-Con). For shRNA experiments, a scrambled non-silencing shRNA (shNC) served as the negative control. Lentivirus particles were prepared in HEK293T cells by co-transfecting (i) psPAX2 (Addgene), (ii) pCMV-VSV-G (Addgene), and (iii) a lentivirus vector in a ratio of 2:1:3. At 72 h after transfection, the lentivirus in culture medium was collected, filtered through 0.45 μm polyvinylidene difluoride filters (Millipore, SLH033) and subsequently stored at −80 °C.

### Cell culture and treatment

HEK293T and A7r5 cell lines were purchased from American type culture collection (ATCC). Human PASMCs (hPASMCs) were purchased from Lonza (Walkersville, MD). HEK293T was cultured in DMEM supplemented with 10% FBS (Cellsera, Australia) and 1% penicillin–streptomycin (Solarbio, P1400) and incubated at 5% CO_2_. Rat pulmonary artery smooth muscle cells (rPASMCs) were isolated from 6-week-old SD male rats as previously described [19] and cultured for 1–2 passages prior to use. rPASMCs were confirmed using immunostaining with anti-α-SMA antibody. rPASMCs and A7r5 cells were grown in DMEM/F12 medium supplemented with 10% FBS and 1% penicillin–streptomycin (Solarbio, P1400), while hPASMCs were cultured in smooth muscle cell medium (ScienCell, Cat. No.1101) containing smooth muscle basal medium, 2% FBS, 1% smooth muscle cell growth supplement (SMCGS, Cat. No. 1152) and 1% penicillin/streptomycin solution (P/S, Cat. No. 0503). For lentiviral infection, 1 × 10^5 cells at 40–50% confluence were infected with 2–3 × 10^5 transduction units (TU) of lentiviruses in the presence of polybrene at a final concentration of 5 μg/mL. When necessary, stable cell lines were selected by treatment with 2 μg/mL puromycin for 1–2 weeks. Primers used and pri-miRNA sequences are listed in Supplementary Table S1 and S5, respectively.

### Quantitative RT-PCR (qRT-PCR)

Total RNA was extracted using RNAiso Plus (TaKaRa, Dalian, China). Mature miRNAs were assessed using the S-Poly(T) method, as previously described [20, 21]. For mRNA assay, the SYBR Green method combined with oligo (dT) plus random primers was used for cDNA synthesis, as previously described [22]. The mRNA expression levels were normalized to β-actin, while miRNA levels were normalized to snoRNA202 (mouse or rat) or snord44 (human). The data were calculated using the 2^−ΔΔCt^ method. Primers used are listed in Supplementary Table S2.

### Western blotting

Animal tissue and cells were lysed with cold RIPA buffer (50 mmol/L Tris·HCl, pH 7.5, 150 mmol/L NaCl, 1% NP-40, 0.25% sodium deoxycholate, and 1 mmol/L EDTA) supplemented with protease inhibitor cocktail (Roche, Mannheim, Germany). Protein concentration was determined using the Bicinchoninic Acid Protein Assay Kit (Thermo Fisher Scientific). Equal amounts of protein (30 μg) were subjected to SDS-PAGE and were subsequently transferred onto PVDF membranes. After blocking with 5% BSA in Tris-buffered saline-Tween 20 (TBST; 20 mmol/L Tris·HCl, pH 7.6, 150 mmol/L NaCl, and 0.1% Tween 20), the membranes were incubated with primary antibodies at 4 °C overnight and then with horseradish peroxidase-conjugated secondary antibodies of goat anti-rabbit IgG or goat anti-mouse IgG at room temperature for 1 h. The protein bands were visualized with a chemiluminescence detection module (Pierce Biotechnology, Rockford, IL) and imaged on a Chemiluminescence Intelligent Image Workstation (BLT GelView 6000Plus). The antibodies used in this study are listed in Supplementary Table S4.

### Wound healing assay

Cell migration was investigated using a wound healing assay. The fully confluent rPASMCs infected with shNC or shMETTL3 lentiviruses and cultured in 24-well plates were scratched. The cells were cultivated in 0.2% FBS and wounded areas were photographed at 0, 48 and 72 h, respectively. The pictures were analyzed using ImageJ and percentage of wound closure was calculated as the formula: migration area (%) = (original area—remaining area)/the original area × 100.

### Cell proliferation assay

The proliferation rate of cells was determined using 5-ethynyl-2′-deoxyuridine (EdU) incorporation with the Cell-Light EdU Apollo567 in Vitro Flow Cytometry Kit (RIBOBIO) according to the manufacturer’s instructions. Briefly, rPASMCs infected with lentiviruses of shMETTL3 or shNC in 48-well plates were incubated with 20 μM EdU reagent for 4 h at 37 °C. Cells were then fixed with paraformaldehyde and permeabilized with 0.5% Triton X-100. DNA was then conjugated with 1 × Apollo reaction cocktail for 30 min, followed by incubation with 1 μg/mL of Hoechst for 10 min. Images were captured under a fluorescent microscope at 10 × magnification.

### Luciferase reporter assays

The regulation of miR-143/145 expression was performed using a dual reporter gene assay, comprising a firefly luciferase construct using pGl4.15 (Promega, E6701) as a backbone, and a reference Renilla luciferase construct pRL-TK (Promega, E2241). A 1.6 kb putative promoter of miR-143/145 was constructed by PCR from rat genomic DNA. The A7r5 cells stably expressing either OE-Con or OE-KLF4 were cotransfected with 500 ng pGl4.15-miR-143/145-WT (or pGl4.15-miR-143/145-Mut) and 50 ng pRL-TK Renilla luciferase reporter using Lipofectamine 3000 (Thermo Scientific). Luciferase activity was measured in cell extracts utilizing a Lumat LB9508 luminometer (Berthold, Bad Wildbad, Germany). The activity of the firefly luciferase in each sample was normalized to the Renilla luciferase activity. Primers used are listed in Supplementary Table S1.

### Hemodynamic measurements

Mean right ventricular systolic pressure (RVSP) was measured using a transonic catheter and subsequently recorded using the MP150 system. This data was analyzed using AcqKnowledge 4.2.0 software package (BIOPAC Systems, Inc.). After the measurements, animals were euthanized. The heart was dissected to evaluate the right ventricular hypertrophy index (RVHI) by calculating the ratio of the weight of RV and that of left ventricle plus ventricular septum (LV + S).

### Tissue preparation and histology

For morphological analysis, lungs embedded in paraffin were sectioned and stained using hematoxylin–eosin. The medial wall thickness was evaluated by examining 10–15 pulmonary arteries from each mouse, with a diameter ranging from 50–100 μm. The percentage of wall thickness and wall area were calculated as the following formulas: relative wall thickness = (outer perimeter—inside perimeter)/outer perimeter, and relative wall area = (outer area—inside area)/outer area, respectively. For immunostaining, lung sections were dewaxed, then rehydrated using a series of alcoholic baths. After antigen retrieval by citric acid buffer (pH 6.0), primary antibodies against α-SMA (1:200, GB111364, servicebio), Calponin (1:200, Abcam, ab46794), METTL3 (1:200, Abcam, ab195352), and Cy3-labeled (1:1000, Jackson ImmunoResearch Labs) or Alexa Fluor 488-labeled secondary antibodies (1:1000, Abcam) were applied. All sections were counterstained with DAPI Fluoromount-G.

### RNA Sequencing and data analysis

Total RNAs were extracted with RNAiso Plus (TaKaRa, Dalian, China). For mRNA transcriptome, cDNA libraries were prepared using the VAHTS Stranded mRNA-seq Library Prep Kit for Illumina (NR612, Vazyme, Inc., Nanjing, China). For small RNA transcriptome analysis, total RNA was electrophoresed through 15% polyacrylamide gels, with fragments ranging from 18 to 30 nt recovered. The library for isolated small RNAs was constructed by using VAHTS Small RNA Library Prep Kit for Illumina (NR801, Vazyme). Libraries were sequenced using the Illumina NovaSeq 6000.

After quality control and mapping the reads to the rat reference genome (RGSC 6.0/rn6), differential expression of the control and treated samples was analyzed using the DESeq2 R package (1.20.0). Gene Ontology (GO) analysis was utilized to determine the potential functions of differentially expressed genes. GO terms with *Q* values ≤ 0.05 were considered to be significantly enriched. Meanwhile, Kyoto Encyclopedia of Genes and Genomes (KEGG) analysis was used to assess the pathways involved differential genes. KEGG terms with *Q* values ≤ 0.05 were considered to be significantly enriched. Both GO and KEGG analyses were performed with KOBAS server.

### miRNA transfection

The Chemically synthesized miRNA mimics or inhibitors for miR-143-3p, miR-145-5p and the corresponding negative control (miR-Con or anti-Con), were acquired from GenePharma (Shanghai, China). rPASMCs at 70% confluence on 10-cm culture dishes were transfected with 400 pmol of either miRNA mimic, mimic control (miR-Con), or miR-143/-145 inhibitors (anti-miR-143, anti-miR-145), inhibitor control (anti-Con) using TurboFect Transfection Reagent (Thermo Scientific). After of transfection, cells were harvested for further analysis at 48 h post-transfection.

### MeRIP-qPCR

Total RNA of rPASMC infected with either shNC or shMETTL3 lentiviruses was subjected to methylated RNA Immunoprecipitation based qPCR (MeRIP-qPCR). Briefly, 10 μg of total RNA was incubated with 1 μg anti-m^6^A antibody (Synaptic Systems, Cat. No. 202 003) or a corresponding control IgG (ab172730, abcam) in 200 μL 1 × IP buffer at 4 °C for 2 h, followed by incubation of protein A/G magnetic beads (GE17152104010150, Merck, USA) at 4 °C for another 2 h. Immunoprecipitated RNA was eluted using Thermolabile Proteinase K (#P8111S, NEB) in reverse transcription buffer, first at 37 °C for 30 min and then at 55 °C for 10 min to inactivate the enzyme. The eluted RNA was directly subjected to RT and qPCR analysis. We also saved 0.5 μg of the same total RNA as input. The m^6^A enrichment in each sample was calculated by normalizing to input.

### RNA methylation quantification

The quantification of RNA methylation was conducted on purified RNA samples using the EpiQuik m^6^A RNA Methylation Quantification Kit (P-9005, EpiGentek), following the manufacturer's instructions.

### Statistical analysis

The data were analyzed using GraphPad Prism version 8.3.0 (GraphPad Software, Inc., San Diego, CA). All data are presented as mean value ± standard deviation (Mean ± SD). The differences between two groups were assessed using a two-tailed unpaired t test, while those among three or more groups were analyzed using one-way ANOVA followed by Tukey's multiple comparisons test. A *P* value less than 0.05 was considered statistically significant.

## Results

### METTL3 protein is upregulated in HPH animals

**Fig. 1:**
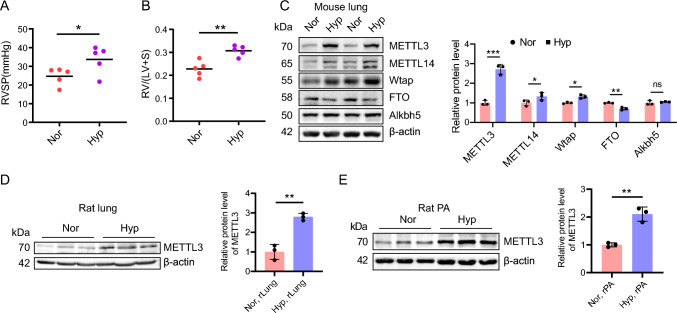
METTL3 protein is upregulated during PH development. **A**, **B** Male C57BL/6 mice (6-week-old) were subjected to either normoxia or hypoxia for 3 weeks. The right ventricular systolic pressure (RVSP) (**A**) and right ventricular hypertrophy index (RVHI) (**B**) for both PH and control animals were assessed (n = 5). **C** The protein levels of METTL3, METTL14, Wtap, FTO and Alkbh5 in the lungs of PH and control animals were detected by western blotting (n = 3). **D**, **E** The protein levels of METTL3 in rat lung (rLung, **D**) and pulmonary artery (rPA, **E**) were also examined by western blotting (n = 3). β-actin was used as a loading control for western blotting. Nor: normoxia; Hyp: hypoxia. Data were analyzed by a two-tailed unpaired t test. Statistical significance is denoted by *P < 0.05, **P < 0.01 and ***P < 0.001. *ns* non-significance

To reveal the role of RNA methylation in the progression of PH, we prepared hypoxic mouse models of PH (Fig. 1A, B), and assessed the expression of key enzymes associated with RNA methylation, including METTL3, METTL14, Wtap, FTO and Alkbh5. Among the three 'writer' proteins, METTL3 was the most significantly upregulated one in the lungs of hypoxic pulmonary hypertension (HPH) mice, while METTL14 and Wtap exhibited smaller increases in expression (Fig. 1C). Oppositely, the m^6^A 'eraser' protein, FTO, was significantly decreased in the HPH mouse lung, whereas Alkbh5 levels remain unchanged. Elevated levels of METTL3 protein were also observed in lung tissues and pulmonary arteries (PAs) of hypoxic PH rats (Fig. 1D, E), indicating the potential regulatory significance of METTL3 in PH progression. Consequently, we focused on METTL3 in following investigation.

### Smooth muscle-specific knockout of *Mettl3* aggravates hypoxia-induced PH in mouse

**Fig. 2:**
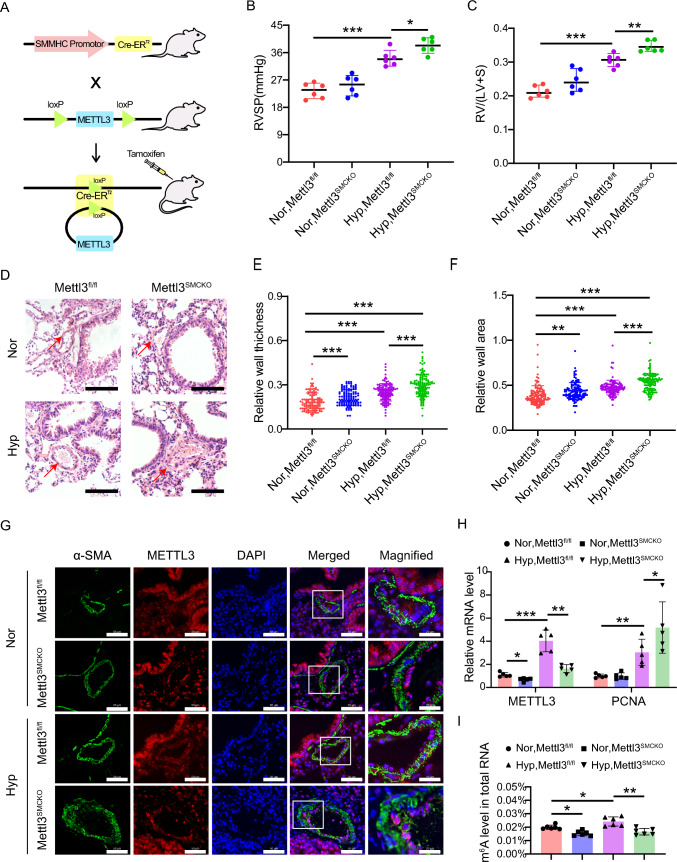
Smooth muscle-specific knockout of *Mettl3* promotes the progression of PH. **A** Schematic illustrating conditional smooth muscle-specific *Mettl3* loss-of-function mouse model. **B**, **C** The RVSP **B** was measured in mmHg by right heart catheterization, and the RVHI **C** was determined as the ratio of the weight of RV to the sum of LV plus ventricular septum (RV/(LV + S)) for knockout mice *SMMHC-Cre*^*ERT2*^*;Mettl3*^*fl/fl*^* (Mettl3*^*SMCKO*^*)* and the control mice *Mettl3*^*fl/fl*^ (n = 6). **D** Representative images of hematoxylin and eosin (HE)-stained lung sections in both *Mettl3*^*SMCKO*^ and *Mettl3*^*fl/fl*^ mice. Scale bars, 100 μm. **E**, **F** The wall thickness of the PAs was calculated for 6 mice, with n = 120 per group. The relative wall thickness was given by (outer perimeter—inside perimeter)/outer perimeter (**E**), and the relative wall area by (outer area—inside area)/outer area (**F**). **G** Representative double-labeled immunostaining with antibodies against METTL3 and α-SMA in the PAs of *SMMHC-Cre*^*ERT2*^*;Mettl3*^*fl/fl*^* (Mettl3*^*SMCKO*^*)* and the control mice *Mettl3*^*fl/fl*^. Scale bars, 50 μm (merged) and 20 μm (magnified). **H** The mRNA levels of METTL3 and PCNA in mouse PAs were evaluated by qRT-PCR (n = 5). β-actin was used as an internal reference for qRT-PCR. **I** The m^6^A levels in total RNA from mouse PAs were analyzed using methylation quantification kit (P-9005, EpiGentek) (n = 6). Nor: normoxia; Hyp: hypoxia. Data were analyzed by using a one-way ANOVA followed by Tukey's multiple comparisons test. Statistical significance is denoted by * *P* < 0.05, ** P < 0.01 and *** P < 0.001

To elucidate the role of METTL3 in PH progression in vivo, we developed smooth muscle-specific *Mettl3* knockout mice (Fig. 2A). The knockout mice of *SMMHC-Cre*^*ERT2*^*;Mettl3*^*fl/fl*^ (*Mettl3*^*SMCKO*^) and the control mice of *Mettl3*^*fl/fl*^ were subjected to either hypoxia (10% O_2_) or normoxia (21% O_2_) exposure for 3 weeks. Hemodynamic analysis revealed that right ventricular systolic pressure (RVSP) and right ventricular hypertrophy index (RVHI) in *Mettl3*^*fl/fl*^ mice were considerably increased under hypoxia compared to those under normoxia (Fig. 2B, C). Moreover, hypoxic *Mettl3*^*SMCKO*^ mice exhibited an even greater elevation in RVSP and RVHI than *Mettl3*^*fl/fl*^ mice under hypoxia (Fig. 2B, C). This suggests that smooth muscle-specific knockout of *Mettl3* exaggerates hypoxia-induced PH hemodynamic changes. In addition, histological analysis revealed a significant augmentation of pulmonary arterial wall thickness and remodeling in *Mettl3*^*SMCKO*^ mice compared to *Mettl3*^*fl/fl*^ mice (Fig. 2D–F). Western blotting analysis confirmed a significant reduction of METTL3 in the PAs of *Mettl3*^*SMCKO*^ mice (Fig. S1), thereby validating the effective knockout of *Mettl3*. Immunostaining also confirmed the lack of METTL3 in the PAs of *Mettl3*^*SMCKO*^ mice, associated with an increase in pulmonary arterial wall thickness, as indicated by anti-α-SMA staining (Figs. 2G, S2). Enhanced pulmonary vascular remodeling in *Mettl3*^*SMCKO*^ mice under both normoxia and hypoxia was further confirmed by anti-Calponin immunostaining, which demonstrated pronounced co-localization with α-SMA (Fig. S2).

The qRT-PCR assay and m^6^A methylation quantification revealed a significant upregulation in METTL3 expression (Fig. 2H) and m^6^A level (Fig. 2I) in the PAs of *Mettl3*^*fl/fl*^ mice under hypoxia compared to normoxia. Conversely, a significant reduction in METTL3 expression and m^6^A level was found in the PAs of *Mettl3*^*SMCKO*^ mice relative to *Mettl3*^*fl/fl*^ mice under both normoxia and hypoxia. Trace amounts of METTL3 were detected in the PAs of knockout mice, potentially originating from tissues such as pulmonary artery endothelial cells (PAECs) and pulmonary artery fibroblasts (PAFs) where METTL3 was not knocked out. Another possibility is the detection of truncated transcripts resulting from exon deletion (including exons 2 and 3, and potentially 4 through alternative splicing) (Figs. S3, S4; Table S3). Sequence analysis revealed that frameshift mutations in these truncated transcripts introduce premature termination codons, precluding the synthesis of functional METTL3 protein. Additionally, the qRT-PCR assay highlighted an elevated level of PCNA in the PAs of *Mettl3*^*SMCKO*^ mice compared to *Mettl3*^*fl/fl*^ mice under hypoxia (Fig. 2H). Furthermore, Ki67 expression significantly increased in both normoxic and hypoxic conditions following *Mettl3* knockout, suggesting that METTL3 plays a broad regulatory role in cell proliferation mechanisms, extending beyond those induced by hypoxia alone (Fig. S5). Overall, our findings suggest that *Mettl3* deletion might aggravate the development of PH in mice, possibly through altering cellular proliferative phenotype.

### METTL3 deficiency drives phenotypic switching in PASMCs

**Fig. 3:**
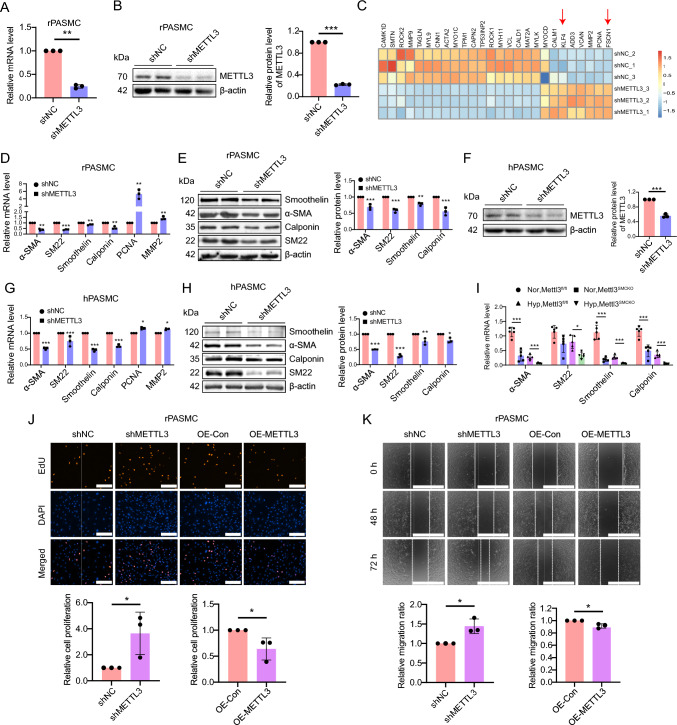
Elimination of METTL3 induces a phenotypic switch in PASMCs from contractile to synthetic. **A**, **B** METTL3 expression levels were assessed in rPASMCs infected with shNC or shMETTL3 lentiviruses by qRT-PCR (**A**) and western blotting (**B**), respectively. The bar chart depicts the relative METTL3 protein level (n = 3). **C** A heatmap displays the expression of VSMCs markers in transcriptome sequencing (n = 3). **D**–**H** The RNA levels of α-SMA, SM22, Smoothelin, Calponin, PCNA and MMP2 in rPASMCs (**D**) and hPASMCs (**G**) infected with shNC or shMETTL3 were determined by qRT-PCR (n = 3). The protein levels of α-SMA, SM22, Smoothelin and Calponin in rPASMCs (**E**) and hPASMCs (**H**) infected with shNC or shMETTL3 were assessed by western blotting (n = 3). The protein levels of METTL3 in shNC or shMETTL3 hPASMCs were assessed (**F**) (n = 3). **I** The mRNA levels of SM22, α-SMA, Smoothelin and Calponin in mouse PAs were determined by qRT-PCR (n = 5). **J** Representative images of EdU labeling depicts the proliferation of rPASMCs upon inhibition and overexpression of METTL3. EdU-positive cells was quantified across 10 random fields, with DAPI staining highlighting all cells (n = 3). Scale bar represents 200 μm. The bar chart illustrates the proportion of EdU-positive cells. **K** Representative images from wound healing assay display the migration of rPASMCs following inhibition and overexpression of METTL3 (n = 3). Scale bar represents 1000 μm. Bar chart elucidates the changes in wound width at 72 h. β-actin was used as an internal reference for qRT-PCR and as a loading control for western blotting. Data were analyzed by a two-tailed unpaired t test, except the expression levels in PAs were analyzed by a one-way ANOVA followed by Tukey's multiple comparisons test. Statistical significance is denoted by * *P* < 0.05, ** P < 0.01 and *** P < 0.001

To clarify the role of METTL3 in influencing PASMCs phenotype, lentivirus-mediated METTL3-specific shRNA was employed to silence METTL3 in rat PASMCs (rPASMCs). Three days after infection, METTL3 expression was dramatically repressed at both mRNA and protein levels in rPASMCs treated with shMETTL3 compared to the shNC group (Fig. 3A, B). Subsequent RNA sequencing (RNA-seq) was performed to analyze the transcriptional shifts in rPASMCs exposed to shMETTL3 versus the control group. Quantitative analysis revealed 1506 differentially expressed transcripts in METTL3-silenced rPASMCs [padj-value < 0.001; fold change (FC) ≥ 2], consisting of 656 up- and 850 down-regulated genes (Fig. S6A). Kyoto encyclopedia of genes and genomes (KEGG) analysis of these differentially expressed genes (DEGs) highlighted pathways impacted by METTL3 inhibition, including vascular smooth muscle contraction, cell adhesion, and calcium signaling (Fig. S6B).

Among the DEGs, contractile marker genes such as α-SMA (Acta2), SM22 (Tagln), Smoothelin (Smtn) and Calponin (Cnn1) were significantly downregulated upon METTL3 inhibition (Fig. 3C). This downregulation was further confirmed by qRT-PCR and western blotting in rPASMCs (Fig. 3D, E) and human PASMCs (hPASMCs) (Fig. 3F–H). Conversely, overexpression of METTL3 (Fig. S7A) led to upregulation of α-SMA, SM22, Smoothelin and Calponin in rPASMCs (Fig. S7B) and hPASMCs (Fig. S7C). Similarly, a downregulation of SM22, α-SMA, Smoothelin and Calponin was observed in the PAs of *Mettl3*^*SMCKO*^ mice relative to *Mettl3*^*fl/fl*^ mice under hypoxia (Fig. 3I). The proliferative marker, PCNA, exhibited heightened levels after METTL3 silencing (Fig. 3D). Simultaneously, the migration marker MMP2 also upregulated following METTL3 inhibition (Fig. 3D) but downregulated with METTL3 overexpression (Fig. S7B-C). In addition, EdU incorporation and wound healing assay further demonstrated that METTL3 knockdown facilitated, whereas its overexpression reduced, the proliferation and migration of rPASMCs (Fig. 3J, K).

Collectively, these findings demonstrate that METTL3 deficiency led to a significant shift in PASMCs from a contractile to a synthetic phenotype, thereby potentiating PH progression.

### Knockdown of METTL3 supresses miR-143/145 expression via m^6^A-dependent impairment of miRNA maturation

**Fig. 4:**
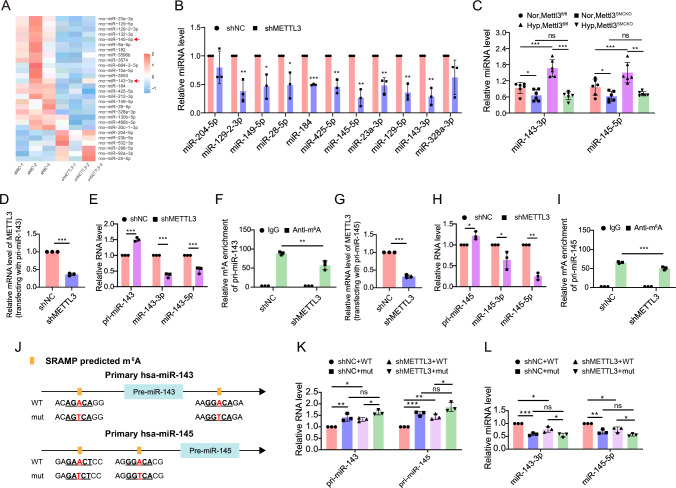
Loss of METTL3 impairs miR-143/145 cluster processing in an m^6^A-dependent manner. **A** A heatmap displays differentially expressed miRNAs in rPASMCs following infection with shNC or shMETTL3 lentiviruses based on small RNA sequencing (n = 3). **B** Differentially expressed miRNAs in rPASMCs were validated by qRT-PCR (n = 3). **C** The expression levels of miR-143-3p and miR-145-5p were detected in the PAs of either *Mettl3*^*SMCKO*^ or *Mettl3*^*fl/fl*^ mice by qRT-PCR (n = 6). snoRNA202 was used as an internal reference in qRT-PCR for miRNA. **D**–**I** HEK293T cells infected with shNC or shMETTL3 lentiviruses were further transfected with pri-miR-143 or pri-miR-145 overexpression plasmids. The inhibition of METTL3 was verified by qRT-PCR (**D**, **G**). The pri-miR-143, miR-143-3p, miR-143-5p (**E**), pri-miR-145, miR-145-3p, and miR-145-5p (**H**) were detected by qRT-PCR (n = 3). The m^6^A enrichment of pri-miR-143 (**F**) or pri-miR-145 (**I**) was ascertained by MeRIP-qPCR (n = 3). **J**–**L** The shNC and shMETTL3 HEK293T cells were transfected with plasmids overexpressing either the wild-type or m^6^A-mutant versions (mut, A-to-T mutation) of human pri-miR-143 and pri-miR-145 (**J**), and the pri-miR-143, miR-143-3p (**K**), pri-miR-145, and miR-145-5p (**L**) were detected by qRT-PCR (n = 3). β-actin or snoRNA202 was used as an internal reference in qRT-PCR for pri-RNA or miRNA (**B**, **C**), respectively. Green fluorescent proteins (GFP) encoded by the pri-miRNA overexpression vector was used as an internal reference in qRT-PCR to evaluate pri-miRNA processing. A two-tailed unpaired t test (**B**–**I**), or one-way ANOVA followed by Tukey's multiple comparisons test (**K**, **L**), were used to estimate the significance. Statistical significance is denoted by * *P* < 0.05, ** P < 0.01 and *** P < 0.001. ns: non-significance

To explore the mechanism by which METTL3 mediates phenotypic transition of PASMCs, small RNA sequencing was conducted in rPASMCs treated with shNC or shMETTL3 lentiviruses. Inhibition of METTL3 resulted in a disordered miRNA expression profile, with a marked decrease of multiple miRNAs including miR-204-5p, miR-129-2-3p, miR-149-5p, miR-28-5p, miR-184, miR-425-5p, miR-145-5p, miR-23a-3p, miR-129-5p, miR-143-3p and miR-328a-3p (Fig. 4A, B). Among them, miR-143-3p and miR-145-5p have been identified as pivotal regulators of VSMCs phenotype [23]. Similarly, we verified that knockout of *Mettl3* markedly reduced the levels of miR-143-3p and miR-145-5p in the PAs of *Mettl3*^*SMCKO*^ mice compared with *Mettl3*^*fl/fl*^ mice under both normoxia and hypoxia conditions (Fig. 4C). Furthermore, METTL3 overexpression resulted in elevated miR-143-3p and miR-145-5p levels in rPASMCs (Fig. S8A) and hPASMCs (Fig. S8B), suggesting that METTL3 plays a regulatory role in the expression of miR-143-3p and miR-145-5p.

We subsequently explore how METTL3 regulates the expression of miR-143-3p and miR-145-5p. Previous studies have reported that METTL3 can modulate pri-miRNA processing through m^6^A modification [24, 25]. To elucidate the role of METTL3 in regulating miR-143/145 maturation, we transfected HEK293T cells, that have been infected with shNC or shMETTL3 lentiviruses, with plasmids overexpressing human pri-miR-143 or pri-miR-145. Our findings demonstrated that METTL3 knockdown increased pri-miR-143 but decreased miR-143-3p and miR-143-5p levels compared with the shNC groups (Fig. 4D, E), suggesting that METTL3 suppression impedes pri-miR-143 processing. Similar results were observed for pri-miR-145 processing upon METTL3 silencing (Fig. 4G, H). MeRIP-qPCR analyses verified that both pri-miR-145 and pri-miR-143 undergo m^6^A methylation (Fig. 4F, I). Moreover, silencing METTL3 led to reduced m^6^A levels in these pri-miRNAs (Fig. 4F, I), suggesting that METTL3-mediated m^6^A reduction impairs the processing of pri-miR-143 and pri-miR-145. Intriguingly, an increase in miR-143-3p and miR-145-5p levels under hypoxic conditions paralleled significant elevations in METTL3 expression in the pulmonary arteries (PAs) of *Mettl3*^*SMCKO*^ mice compared with *Mettl3*^*fl/fl*^ mice (Figs. 4C, 2H). Additionally, depletion of *Mettl3* led to high levels of pri-miR-143 and pri-miR-145 in the PAs of *Mettl3*^*SMCKO*^ mice compared to *Mettl3*^*fl/fl*^ mice under both normoxic and hypoxic conditions (Fig. S9). These results underscore the role of METTL3 in regulating the production of miR-143-3p and miR-145-5p.

Next, we employed SRAMP (http://www.cuilab.cn/sramp/) for further analysis and identified potential m^6^A modification sites within both pri-miR-143 and pri-miR-145 in human, rat and mouse (Figs. 4J, S10, S11, and S12; Supplementary Table S5). To investigate the effects of m^6^A modifications on miRNA processing, we generated plasmids overexpressing human pri-miR-143 and pri-miR-145 with adenosines at these sites replaced by thymines (Fig. 4J). Our experiments revealed that mutation at these m^6^A sites resulted in effects akin to those observed with METTL3 inhibition: increased levels of pri-miR-143 but decreased miR-143-3p levels compared with the wild-type (WT) controls (Fig. 4K, L). Similar results were observed for pri-miR-145, indicating that METTL3-mediated m^6^A modification plays a crucial role in the processing of pri-miR-143 and pri-miR-145.

Previous studies have identified hnRNPA2B1 as an m^6^A mediator during miRNA maturation, and its inhibition impedes miRNA processing [26]. Our results showed that silencing hnRNPA2B1 significantly reduced the expression of miR-143-3p and miR-145-5p (Fig. S13A, B), whereas overexpression of hnRNPA2B1 increased their levels (Fig. S13C, D). Silencing METTL3 reversed the hnRNPA2B1-induced enhancement of miR-143-3p and miR-145-5p expression (Fig. S13D), indicating a potential role for hnRNPA2B1 in m^6^A-mediated processing of miR-143/145 cluster.

In summary, our findings demonstrate that inhibiting METTL3 reduces miR-143/145 levels through m^6^A-dependent disruption of miRNA maturation. Furthermore, hypoxia-induced upregulation of METTL3, along with the subsequent increase in miR-143/145, may protect against the development of PH.

### miR-145-5p and miR-143-3p regulate phenotypic transformation of PASMCs via their specific targets KLF4 and FSCN1

**Fig. 5:**
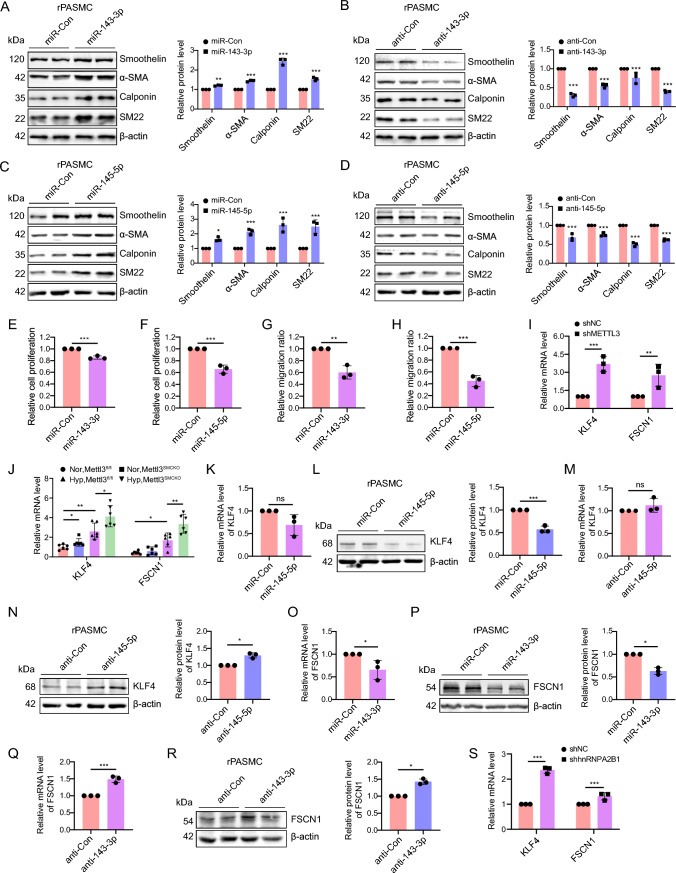
miR-145-5p and miR-143-3p modulate PASMCs phenotypic switching via their specific targets. **A**–**D** Western blotting analysis was conducted to determine the expression of SM22, α-SMA, Smoothelin, and Calponin in rPASMCs transfected with miR-143-3p mimic (**A**), miR-143-3p inhibitor (**B**), miR-145-5p mimic (**C**) and miR-145-5p inhibitor (**D**) (n = 3). Bar charts represent the relative protein levels. **E**–**H** Transfection of miR-143-3p and miR-145-5p mimics was performed in rPASMCs, followed by EdU incorporation and wound healing assay. Bar chart illustrating the proportion of EdU-positive cells upon transfection of miR-143-3p (**E**) and miR-145-5p (**F**) (n = 3). Bar chart also elucidates the changes of wound width at 72 h in wound healing assay upon transfection of the mimics of miR-143-3p (**G**) and miR-145-5p (**H**). **I** The expression levels of KLF4 and FSCN1 in METTL3-silenced rPASMCs were assessed by qRT-PCR (n = 3). **J** The mRNA levels of KLF4 and FSCN1 in mouse PAs were determined by qRT-PCR (n = 6). **K**–**N** The KLF4 mRNA and protein levels in rPASMCs transfected with miR-145-5p mimic (**K**–**L**) and miR-145-5p inhibitor (**M**–**N**) were detected by qRT-PCR and western blotting, respectively (n = 3). **O**–**R** Similarly, the FSCN1 mRNA and protein levels in rPASMCs transfected with miR-143-3p mimic (**O**–**P**) and miR-143-3p inhibitor (**Q**–**R**) were also assessed using the same techniques (n = 3). **S** The expression levels of KLF4 and FSCN1 in hnRNPA2B1-silenced rPASMCs were assayed by qRT-PCR (n = 3). β-actin was used as an internal reference for qRT-PCR and as a loading control for western blotting. A two-tailed unpaired t test (**A**–**I**, **K**–**S**), or one-way ANOVA followed by Tukey's multiple comparisons test (**J**), were used to estimate the significance. Statistical significance is denoted by * *P* < 0.05, ** P < 0.01 and *** P < 0.001. ns: non-significance

To determine the METTL3-mediated role of miR-143/145 in PASMCs phenotypic modulation, rPASMCs were transfected with miR-143-3p or miR-145-5p mimic and inhibitor. The data indicated that introduction of miR-143-3p or miR-145-5p mimic upregulated contractile markers such as Smoothelin, α-SMA, Calponin and SM22 (Fig. 5A, C). In contrast, their inhibitors led to downregulation of these contractile proteins (Fig. 5B, D). Introduction of miR-143-3p or miR-145-5p mimic yielded similar effects in hPASMCs as observed in rPASMCs (Fig. S14A, B), highlighting the pivotal role of miR-143-3p and miR-145-5p in influencing the PASMC phenotype. The EdU assay demonstrated that transfection with either miR-143-3p or miR-145-5p mitigated the cellular proliferation in both rPASMCs (Figs. 5E, F, S15A) and hPASMCs (Fig. S15B, C). Furthermore, a wound healing assay indicated that the introduction of miR-143-3p or miR-145-5p mimic suppressed the migration of rPASMCs, further validating their functional importance in modulating PASMC behavior (Figs. S15D, 5G, H).

To discern the molecular mechanisms through which miR-143/145 influence PASMCs phenotype, we aimed to identify their targets. Transcriptome analysis, followed by qRT-PCR assays, revealed a significant upregulation of Krüppel-like Factor 4 (KLF4) and fascin actin-bundling protein 1 (FSCN1) in METTL3-silenced rPASMCs (Figs. 3C, 5I). Remarkably, depletion of *Mettl3* markedly resulted in a pronounced elevation of KLF4 and FSCN1 expression levels in the PAs of *Mettl3*^*SMCKO*^ mice compared to *Mettl3*^*fl/fl*^ mice under hypoxic conditions (Fig. 5J). Previous studies identified KLF4 as a target of miR-145-5p in human embryonic stem cells [27] and FSCN1 as a target of miR-143-3p in esophageal squamous cell carcinoma [28] (Fig. S16). In rPASMCs, we observed that the miR-145-5p mimic reduced the protein levels of KLF4 without affecting its mRNA levels (Fig. 5K, L), whereas its inhibitor increased the protein levels of KLF4 without altering its mRNA levels (Fig. 5M, N). Similarly, the miR-143-3p mimic decreased both mRNA and protein levels of FSCN1 (Fig. 5O, P), whereas its inhibitor enhanced them (Fig. 5Q, R). This consistent negative correlation confirmed the direct targeting of KLF4 by miR-145-5p and FSCN1 by miR-143-3p, highlighting their roles in regulating the PASMC phenotype.

The significant reduction in miR-143/145 expression following hnRNPA2B1 silencing led us to propose a regulatory role for hnRNPA2B1 on KLF4 and FSCN1 expression. Confirming our hypothesis, hnRNPA2B1 knockdown resulted in elevated levels of KLF4 and FSCN1 (Figs. S13A, 5S), underscoring the impact of hnRNPA2B1 on the biogenesis of miR-143/145.

Taken together, the results suggest that RNA methylation-mediated modulation of miR-143/145 cluster plays an essential role in PASMCs phenotypic transformation via specific targets.

### A miR-143/145-KLF4 positive feedback loop facilitates PASMCs phenotypic transition

**Fig. 6:**
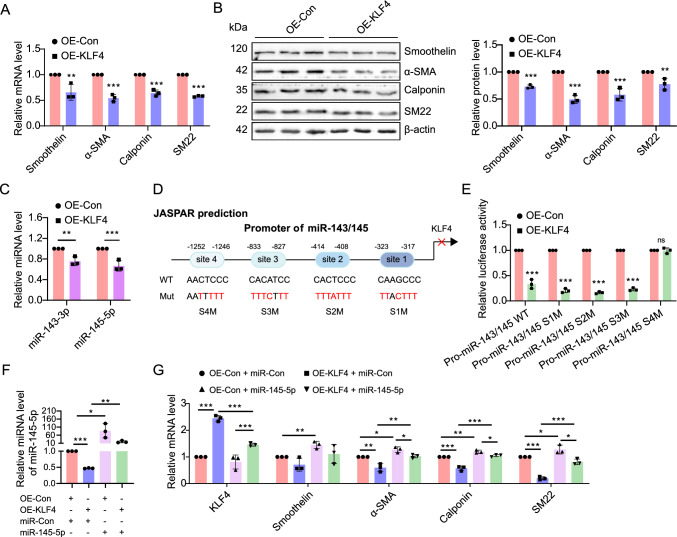
A positive feedback circuit between miR-143/145 and KLF4 promotes PASMCs phenotypic switching. **A**, **B** The mRNA and protein levels of SM22, α-SMA, Smoothelin and Calponin in rPASMCs transfected with OE-Con or OE-KLF4 lentiviruses were measured by qRT-PCR (**A**) and western blotting (**B**), respectively (n = 3). Bar charts show the relative protein levels. β-actin was used as an internal reference for qRT-PCR and as a loading control for western blotting. **C** The miR-143-3p and miR-145-5p levels were detected in OE-Con or OE-KLF4 rPASMCs by qRT-PCR (n = 3). snoRNA202 was used as an internal reference in qRT-PCR. **D** The potential binding site of KLF4 on miR-143/145 promoter was predicted using the JASPAR database (http://jaspar.genereg.net/). **E** The OE-Con or OE-KLF4 A7r5 cells were transfected with the luciferase reporter plasmids containing the wild-type miR-143/145 promoter or promoter with indicated mutation (site 1, − 317 to − 323; site 2, − 408 to − 414; site 3, − 827 to − 833; site 4, − 1246 to − 1252). The relative luciferase activity was measured at 48 h post-transfection (n = 3). **F**, **G** The expression levels of miR-145-5p (**F**), as well as KLF4, Smoothelin, SM22, α-SMA and Calponin (**G**) were detected by qRT-PCR in OE-Con or OE-KLF4 rPASMCs transfected with miR-145-5p mimic or its control (miR-Con) (n = 3). A two-tailed unpaired t test (**A**–**D**), or one-way ANOVA followed by Tukey's multiple comparisons test (**F**, **G**), were used to estimate the significance. Statistical significance is denoted by * *P* < 0.05, ** P < 0.01 and *** P < 0.001

Previous studies have highlighted the key role of KLF4 in regulating phenotypic switching in smooth muscle cells [29, 30]. Our findings reinforced this by showing that KLF4 overexpression decreased the mRNA and protein levels of contractile markers, including SM22, α-SMA, Calponin and Smoothelin in rPASMCs (Fig. 6A, B).

Previous evidence has indicated that SRF/Myocd binds to the CArG cis-element on miR-143/145 promoter, enhancing miR-143/145 expression [15, 18, 23]. Inversely, KLF4 diminishes the binding affinity of serum response factor (SRF) to CArG-box elements [30–33]. Given these observations, we proposed that KLF4 might negatively regulate miR-143/145 expression at transcriptional level. Substantiating our hypothesis, we observed marked decreases in miR-143-3p and miR-145-5p levels upon KLF4 overexpression in rPASMCs (Fig. 6C). Using the JASPER database, four potential KLF4 binding sites on the promoter region of miR-143/145 were predicted (Fig. 6D). We then constructed the miR-143/145 promoter into a luciferase reporter and found that KLF4 overexpression significantly repressed the luciferase activity compared with the control group (Fig. 6E). Nevertheless, mutation of binding site 4, but not site 1, 2 and 3, abolished the suppressive effect of KLF4 on the reporter activity (Fig. 6E), confirming that KLF4 targets the miR-143/145 promoter at predicted site 4.

To further ascertain the bidirectional regulatory relationship between KLF4 and miR-145-5p, we conducted a rescue experiment by introducing either mimic control or miR-145-5p mimic into KLF4 overexpressed rPASMCs (Fig. 6F). Our results revealed that miR-145-5p overexpression reversed the KLF4-induced reduction in contractile genes (Figs. 6G, S17), highlighting the role of KLF4 in shaping the PASMCs phenotype through miR-145-5p. In sum, we illuminate a sophisticated positive feedback loop between KLF4 and miR-143/145, wherein METTL3-guided m^6^A methylation orchestrates PASMCs phenotypic switching.

**Fig. 7:**
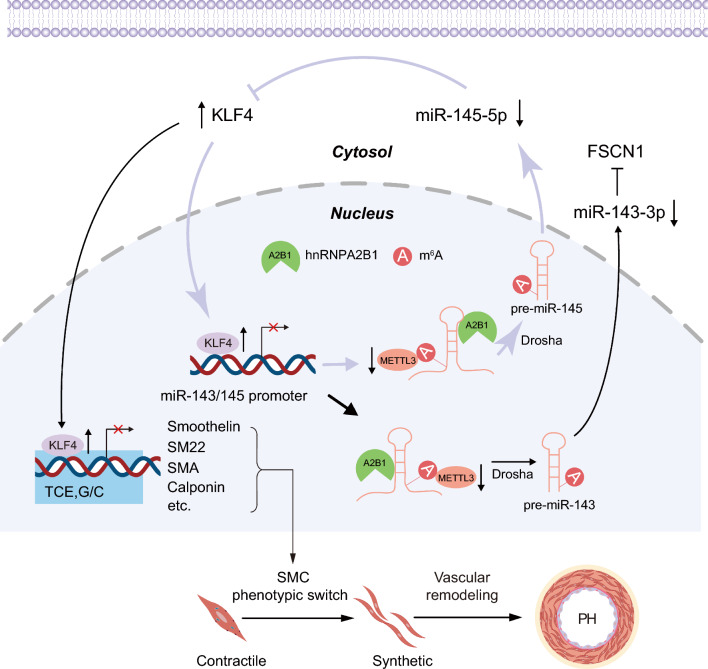
Within PASMCs, METTL3 depletion reduces m^6^A modification, subsequently diminishing miR-143/145 expression via impeding miRNA processing in an m^6^A-dependent manner. The reduction in mature miR-145-5p and miR-143-3p enhances their respective targets, KLF4 and FSCN1. Augmented KLF4 in turn represses the transcription of miR-143/145 cluster, establishing a positive feedback loop with miR-143/145. This perpetuating cycle suppresses contractile markers, facilitating the phenotypic switch in PASMCs and pulmonary vascular remodeling. Hypoxia-induced upregulation of METTL3 and consequent increase of miR-143/145 may serve as a compensatory and protective agent against the progression of PH

Taken together, the current research reveals a novel epigenetic regulatory mechanism influencing miR-143/145 cluster expression in phenotypic switch of PASMCs. METTL3-driven and hnRNPA2B1-mediated m^6^A modification play a key role in regulating miR-143/145 cluster. METTL3 reduction mitigates miR-143/145 via m^6^A-dependent impairment of miRNA maturation. A miR-143/145-KLF4 positive feedback loop potentiates the repression of contractile markers genes, facilitating PASMCs phenotypic transition (Fig. 7). Our findings unmask a promising therapeutic approach via targeting m^6^A modified miR-143/145-KLF4 loop for PH treatment.

## Discussion

Pulmonary hypertension (PH) is a progressive vascular lung disease characterized by vascular remodeling primarily attributed to phenotypic transformation of PASMCs [1, 34–36]. However, the underlying mechanisms of PASMCs dysregulation in PH are not fully understood.

Accumulating evidence suggests that RNA methylation plays a crucial role in PASMCs phenotypic switching. METTL14 mediated m^6^A methylation leads to mRNA decay of Grb-2-related adaptor protein (GRAP), thus promoting the proliferation and migration of hypoxic human PASMCs via Ras/ERK signaling pathway [37]. Similarly, YTHDF1 identified the m^6^A mark on Foxm1 mRNA, facilitating hypoxia-induced PASMCs proliferation and the expression of proliferative markers [38]. Conversely, deletion of YTHDF1 alleviated phenotype switch of PASMCs through recognizing m^6^A modified MAGED1 mRNA [10]. Our experiments confirmed that METTL3 was upregulated in lungs and PAs of PH animal. METTL3 depletion induced a marked phenotypic switch in PASMCs in vitro and enhanced pulmonary vascular remodeling in vivo. The deficiency of METTL3 significantly mitigated the expression of miR-143/145 cluster and elevated the levels of their target genes, FSCN1 and KLF4. The reduction of miR-143/145 upon METTL3 deficiency is due to the impediment of m^6^A-mediated miRNA processing. Consequently, our findings reveal the essential role of METTL3-mediated m^6^A modification on miRNA-143/145 in phenotypic switching of PASMCs and PH progression.

Many studies suggest that miR-143/145 play a key role in maintaining contractile activity of VSMCs [14–17]. In this study, miR-143/145 exhibit a pronounced elevation in hypoxic mouse PAs. Furthermore, miR-143/145 are regulated by METTL3 and play crucial roles in PASMCs under normoxic and hypoxic conditions. The introduction of miR-143/145 maintains the contractile phenotype of PASMCs. Additionally, these miRNAs regulate cell proliferation and migration, with miR-145-5p directly targeting KLF4, and miR-143-3p targeting FSCN1. These findings highlight the importance of miR-143-3p and miR-145-5p in PASMC function and their potential involvement in PH and vascular diseases.

Mechanistically, METTL3 deficiency decreases the level of m^6^A modification on pri-miR-143/145, impeding miR-143/145 maturation and leading to significant downregulation of mature miR-143/145. Multiple reports have described the effects of m^6^A on miRNA processing. For instance, METTL3 silencing inhibited m^6^A level on pri-miR-375, reducing miR-375-3p expression [39]. Peng et al. demonstrated that KIAA1429/ALKBH5-mediated m^6^A modifications control the processing of pri-miR-143-3p through interacting with the microprocessor protein DGCR8 [40]. In human bronchial epithelial 16HBE cells, METTL3 silencing led to reduced m^6^A modification and miR-143-3p expression, thus promoting airway epithelial cells epithelial mesenchymal transition (EMT) and increasing the production of extracellular matrix (ECM) in lung fibroblasts through targeting Smad3 [41]. We, therefore, conclude that METTL3 is capable of controlling the expression of a series of miRNAs including miR-143/145 cluster in an m^6^A-dependent manner, and that hypoxia-induced METTL3 upregulation, along with the increase of miR-143/145, could serve as a protective mechanism against PH progression under hypoxia.

Transcription factor KLF4 is instrumental in controlling VSMC phenotypic switching [12, 29, 32, 42, 43]. Through binding to the G/C repressive element and the transforming growth factor-β (TGF-β) control element (TCE), KLF4 significantly decreases contractile marker genes in VSMCs [31, 44]. In this study we unmask that genetic deletion and silencing of METTL3 significantly upregulate KLF4 in rPASMCs. Enhanced KLF4 represses contractile proteins, resulting in a remarkable shift of PASMCs phenotype. We verified that KLF4 can be directly targeted by miR-145-5p in rPASMCs, resembling findings in human embryonic stem cells [27]. Using a luciferase reporter assay, we demonstrate that KLF4 binds to the promoter of miR-143/145 cluster and represses its transcriptional activity, thereby establishing a positive feedback loop between KLF4 and miR-143/145. While KLF4 has been reported to inhibit miR-143 transcription by binding to its upstream motifs [45], to our knowledge, this study pioneered the identification of a feedback loop between KLF4 and miR-143/145 cluster. Similarly, miR-145 and its target, the transcription factor OCT4, form a double-negative feedback loop that modulates the transition between self-renewal and differentiation in human embryonic stem cells (hESCs) [27]. We therefore hypothesize that this regulatory circuit involving miR-143/145 and its transcription factor target could represent a fundamental mechanism governing cellular phenotype transformation.

Contrary to our findings, Qin et al. reported that increased METTL3 under hypoxia enhanced m^6^A-mediated degradation of PTEN, promoting PASMC proliferation and migration [46]. The discrepancy may arise from differences in experimental models and METTL3 targets. Our study used a SMC-specific *Mettl3* knockout model to investigate METTL3 effects on PASMCs in vivo, while Qin's study used wild-type rats under hypoxia, reflecting outcomes involving various cell types such as PASMCs, PAECs, and PAFs. Additionally, we focused on the role of METTL3 in miR-143/145 expression, whereas Qin's study examined PTEN modulation, potentially resulting in different PASMC phenotypes. In line with our findings, Lin et al. reported that METTL3 inhibition in adipose-derived stem cells (ADSCs) suppressed the expression of VSMC-specific marker genes such as α-SMA, SM22, Calponin, and SMMHC [8], suggesting a protective role of METTL3 in preserving the differentiated phenotype of VSMCs. Collectively, these findings emphasize the context-dependent and target-specific effects of METTL3 in biological process and diseases like PH.

Consistent with our observations, Caruso et al. [47] and Deng et al. [48] have also reported elevated expression of miR-143 and miR-145 in hypoxic animal models of PH. However, their studies concluded that miR-143 and miR-145 have detrimental effects on PH progression, contrasting to our findings. This divergence may be attributable to variations in experimental models and techniques, the involvement of different cell types in PH, and the specific downstream targets and signaling pathways modulated by miR-143/145. Our investigation employed the SMC-specific *Mettl3* knockout approach to specifically elucidate the roles of miR-143 and miR-145 in the smooth muscle layer in PH. In contrast, Caruso and Deng utilized global knockout models and administered anti-miRs subcutaneously in animals. Consequently, their studies may reflect the combined effects of miR-143 and miR-145 on various cell types involved in PH, including PASMCs, PAECs and PAFs. Additionally, our results suggest that miR-145 and miR-143 influence PH development through their impact on KLF4 and FSCN1, respectively. In contrast, Caruso's study implicated the Wnt/β-catenin signaling pathway in the action of miR-145, whereas Deng's study did not identify a specific target for miR-143. The variability in downstream targets and pathways modulated by miR-143 and miR-145 could result in differing outcomes regarding their impact on PH. Similar inconsistency in miR-143/145 functionality is also observed in atherosclerosis research. Overexpression of miR-145 exacerbates inflammation in cell and animal models of atherosclerosis through NF-κB pathway activation, suggesting a detrimental impact on the disease [49, 50]. However, miR-145 expression is reduced in human atherosclerotic aortas, associated with decreased SMC contractile markers such as Calponin, α-SMA, and myocardin, implying that loss of miR-145 may exacerbate atherosclerosis [51]. Taken together, these discrepancies underscore the complexity and dynamic nature of miRNA regulation in cardiovascular diseases. A comprehensive understanding of the context-dependent effects, downstream regulatory pathways, epigenetic and post-genomic modulation of miR-143/145 in SMC proliferation, arterial remodeling, and inflammation is essential to unravel the intricate molecular mechanisms underlying PH and other vascular diseases [52].

Claudio et al. reported that hnRNPA2B1 functions as an m^6^A reader in pri-miRNAs, enhancing their maturation into mature miRNAs [26]. However, Wu et al. later revealed through crystal structure analysis of hnRNPA2B1 with various RNA substrates that m^6^A likely increases the accessibility of hnRNPA2B1 to pri-miRNAs rather than directly binding to it, thereby supporting pri-miRNAs maturation [53]. Our research corroborates the vital role of hnRNPA2B1 as an m^6^A mediator in miRNA processing, demonstrating that its deficiency leads to impaired maturation of pri-miR-143/145.

In this study, we use whole lung tissue instead of isolated PASMCs for assessing *Mettl3* knockout in mRNA transcripts, as depicted in Figure S4B. This approach was chosen due to the challenges associated with isolating PASMCs from small mouse PAs. Future research incorporating isolated PASMCs from *Mettl3* knockout mice will provide clearer insights and reduce confounding influences from other cell types, as recommended by established protocols [54]. Additionally, we conducted transcriptome analysis and qRT-PCR assays to identify potential targets of miR-143/145, such as KLF4 and FSCN1, in METTL3-silenced rPASMCs and *Mettl3*^*SMCKO*^ mice under hypoxic conditions. While these approaches have provided valuable insights, we recognize that proteomic methods, including mass spectrometry and advanced protein arrays, would offer a more direct assessment of the impact of miR-143/145 on protein expression and could reveal additional targets that may be overlooked in transcriptomic analyses. It is worth noting that proteomic analysis might also have limitations in detecting proteins with low expression abundance. Future studies could benefit from integrating proteomic approaches with transcriptomic analyses to obtain a comprehensive understanding of the molecular mechanisms through which miR-143/145 influence PASMC phenotype.

## Conclusion

This study unveils a previously unidentified m^6^A-regulated miR-143/145-KLF4 positive feedback circuit essential for determining the PASMCs phenotype. Our insights highlight a potential therapeutic avenue by targeting the m^6^A-modification pathway linked to miR-143/145 cluster for PH management.

### Supplementary Information

Below is the link to the electronic supplementary material.

Supplementary file1 (DOC 13436 KB)

## Data Availability

NGS data have been deposited in NCBI Sequence Read Archive (SRA) and are available through SRA accession number PRJNA1018125.

## Abbreviations

m^6^A: N6-methyladenosine
FSCN1: Fascin actin-bundling protein 1
HPH: Hypoxic pulmonary hypertension
KLF4: Krüppel-like factor 4
LV: Left ventricle
miRNAs: MicroRNAs
PA: Pulmonary artery
PAECs: Pulmonary artery endothelial cells
PAFs: Pulmonary artery fibroblasts
PASMCs: Pulmonary artery smooth muscle cells
PH: Pulmonary hypertension
RV: Right ventricle
RVHI: Right ventricular hypertrophy index
RVSP: Right ventricular systolic pressure
VSMCs: Vascular smooth muscle cells
MeRIP: Methylated RNA Immunoprecipitation
