# Supplementary material for: N6-methyladenosine-driven miR-143/145-KLF4 circuit orchestrates the phenotypic switch of pulmonary artery smooth muscle cells
Source: Cell Mol Life Sci. 2024 Jun 12;81(1):256. doi: 10.1007/s00018-024-05304-1 (PMC11335293; doi:10.1007/s00018-024-05304-1)
Supplement: Supplementary file 1 — Supplementary file1 (DOC 13436 KB) [file 18_2024_5304_MOESM1_ESM.doc]

**N6-methyladenosine-guided miR-143/145-KLF4 circuit** **orchestrates the phenotypic switch of pulmonary artery smooth muscle cells**

Kang Kang^1#^, Chuannan Sun^1#^, Hui Li^1^, Xiaojia Liu^1^, Jingyuan Deng^1^, Silei Chen^1^, Le Zeng^2^, Jiahao Chen^1^, Xinyi Liu^1^, Jiahao Kuang^1^, Jingjing Xiang^1^, Jingqian Cheng^1^, Xiaoyun Liao^2^, Mujin Lin^1^, Xingshi Zhang^1^, Chuzhi Zhan^1^, Sisi Liu^1^, Jun Wang^2^, Yanqin Niu^2^, Cuilian Liu^2^, Cai Liang^2^, Jinsheng Zhu^3^, Shuxin Liang^3^, Haiyang Tang^3^ and Deming Gou^2*^

Data Supplement

1. Supplementary Table S1
2. Supplementary Table S2
3. Supplementary Table S3
4. Supplementary Table S4
5. Supplementary Table S5
6. Supplementary Figure S1
7. Supplementary Figure S2
8. Supplementary Figure S3
9. Supplementary Figure S4
10. Supplementary Figure S5
11. Supplementary Figure S6
12. Supplementary Figure S7
13. Supplementary Figure S8
14. Supplementary Figure S9
15. Supplementary Figure S10
16. Supplementary Figure S11
17. Supplementary Figure S12
18. Supplementary Figure S13
19. Supplementary Figure S14
20. Supplementary Figure S15
21. Supplementary Figure S16
22. Supplementary Figure S17

Supplementary Table S1

Primers used for plasmid construction

| **Name** | **Sequence (5′-3′)** | **Note** |
| --- | --- | --- |
| XhoI r miR-143/145 Pro F1 | 5'-TAGCCTCGAGCCCAGTTTGTAGATGGGGAATG-3' | pGl4.3-rmiR-143/145 Pro-luc |
| MluI r miR-143/145 Pro R1 | 5'-TAGCACGCGTTGGCACCCCTTGATGCTACAG-3' |
| miR -143/145-KLF4-BS1M-F | 5'-GGCAACTCTCAGAAATCCGTCCCCTTTATTTTTCCTCCTCTCCTCCCTGCCTCCT-3' |
| miR -143/145-KLF4-BS1M-R | 5'-AGGAGGCAGGGAGGAGAGGAGGAAAAATAAAGGGGACGGATTTCTGAGAGTTGCC-3' |
| miR -143/145-KLF4-BS2M-F | 5'-CACATGGGCTCTGGGGGCTGAAGCTTACTTTTCAGCATTGTCTGAAACCTCCAAA-3' |
| miR -143/145-KLF4-BS2M-R | 5'-TTTGGAGGTTTCAGACAATGCTGAAAAGTAAGCTTCAGCCCCCAGAGCCCATGTG-3' |
| miR -143/145-KLF4-BS3M-F | 5'-CCAACAGAGCAGGAGCAGGCCTTGGACTAAAGAAAGCGAAGAATACCTGCAAACTCT-3' |
| miR -143/145-KLF4-BS3M-R | 5'-AGAGTTTGCAGGTATTCTTCGCTTTCTTTAGTCCAAGGCCTGCTCCTGCTCTGTTGG-3' |
| miR -143/145-KLF4-BS4M-F | 5'-GGAAACCTGGGTAGCTGTAAAAATTGCCCAGCCCGGAGCCT-3' |
| miR -143/145-KLF4-BS4M-R | 5'-AGGCTCCGGGCTGGGCAATTTTTACAGCTACCCAGGTTTCC-3' |
| EcoRI hMETTL3-F1 | 5'-ATCGCGTCTCGAATTCATGTCGGACACGTGGAGC-3' | OE human METTL3 |
| BamHI hMETTL3-R1 | 5'-CGATCGTCTCGGATCCCTATAAATTCTTAGGTTTAG-3' |
| EcoRI rat KLF4 F1 | 5'-ACTAGAATTCATGAGGCAGCCACCTGGCGA-3' | OE rat KLF4 |
| BglII rat KLF4 R1 | 5'-CGATAGATCTTTAAAAGTGCCTCTTCATGTGTAAGGCA-3' |
| XhoI pri-miR-143 F | 5'-CACCCTCGAGTCCTCCTTCTAAATTAAGC-3' | OE human pri-miR-143 |
| EcoRI pri-miR-143 R | 5'-GAGAATTCACAGAGGAGTCAGGACATG-3' |
| XhoI pri-miR-145-F | 5'-CACCTCGAGGAATACACATGAGCCGTGCAG-3' | OE human pri-miR-145 |
| EcoRI pri-miR-145 R | 5'-GAGAATTCCAGCCTCACAGGGATGTTATG-3' |
| shmrMETTL3-P1 | 5'-ACCGCGTCAGTATCTTGGGCAAATTCTCGAGAAT-3' | Construct lenti-shRNA for METTL3 in mouse or rat |
| shmrMETTL3-P2 | 5'-AAAACGTCAGTATCTTGGGCAAATTCTCGAGAAT-3' |
| shmrMETTL3-P3 | 5'-TTGCCCAAGATACTGACG-3' |
| shhMETTL3-P1 | 5'-ACCGCGTCAGTATCTTGGGCAAGTTCTCGAGAAC-3' | Construct lenti-shRNA for METTL3 in human |
| shhMETTL3-P2 | 5'-AAAACGTCAGTATCTTGGGCAAGTTCTCGAGAAC-3' |
| shhMETTL3-P3 | 5'-TTGCCCAAGATACTGACG-3' |
| rshHNRNPA2B1 P1 | 5'-ACCGGTCACAATGCAGAAGTTAGAACTCGAGTTC-3' | Construct lenti-shRNA for hnRNPA2B1 in rat |
| rshHNRNPA2B1 P2 | 5'-AAAAGTCACAATGCAGAAGTTAGAACTCGAGTTC-3' |
| rshHNRNPA2B1 P3 | 5'-TAACTTCTGCATTGTGAC-3' |
| SMMHC-CreERT2 F1 | 5'-TGACCCCATCTCTTCACTCC-3' | mouse genotype identification |
| SMMHC-CreERT2 R1 | 5'-AGTCCCTCACATCCTCAGGTT-3' |
| Mettl3-loxP F1 | 5'-CAAACCTTTACTGTGCTTCCATGA-3' |
| Mettl3-loxP R1 | 5'-ATAAACACCAGGCCCTTGGAATAC-3' |
| Mettl3-loxP F2 | 5'-CCTCCTTCCCCAGATGAAACTGT-3' |
| Mettl3-loxP R2 | 5'-CGTCATCGAAGACCAAACTAACAT-3' |

Supplementary Table S2

qPCR Primers

| **Name** | **Sequence (5′-3′)** |
| --- | --- |
| hrmMETTL3-F1 | 5'-CCAGGAGCTTGCTCTTACACA-3' |
| hrmMETTL3-R1 | 5'-GGTCAGCCATCACAACTGCA-3' |
| β-actin-F | 5'-AGAGATGGCCACGGCTGCTT-3' |
| β-actin-R | 5'-ATTTGCGGTGGACGATGGAG-3' |
| mr Beta-actin F | 5'-GTGACGTTGACATCCGTAAAGA-3' |
| mr Beta-actin R | 5'-GCCGGACTCATCGTACTCC-3' |
| rat Smoothelin-F | 5'-CACTCTCAACAGCGAGAAGCC-3' |
| rat Smoothelin-R | 5'-GGATCAACTTGCGTTCCTCGT-3' |
| rat α-SMA-F | 5'-ATCCGATAGAACACGGCATCA-3' |
| rat α-SMA -R | 5'-TAGCAAGGTCGGATGCTCCTC-3' |
| rat Calponin-F | 5'-ATTGGCCTACAGATGGGCAC-3' |
| rat Calponin-R | 5'-TCAAAGATCTGCCGCTTGGT-3' |
| rat SM22-F | 5'-ACTACCGTGGAGATCCCAA-3' |
| rat SM22-R | 5'-TTTCTAACTGATGATCTGCC-3' |
| rat MMP2-F | 5'-ACTTTGGTCGATGGGAGCAT-3' |
| rat MMP2-R | 5'-GCTGCTGTATTCCCGACCAT-3' |
| rat PCNA-F | 5'-GTGAAGTTTTCTGCGAG-3' |
| rat PCNA-R | 5'-GAGACAGTGGAGTGGCT-3' |
| rat FSCN1-F | 5'-GACTGCGAAGGTCGCTACC-3' |
| rat FSCN1-R | 5'-CTGATCCGTCTCTTCATCCTGA-3' |
| rat KLF4-F | 5'-ACGAAGAGTTCTCATCTCAAGG-3' |
| rat KLF4-R | 5'-GTAGTGCCTGGTCAGTTCATC-3' |
| mouse Smoothelin-F | 5'-ATGGCAGACGAGGCTTTAGC-3' |
| mouse Smoothelin-R | 5'-AGTGTAGCCAGTTCTCCTTGTT-3' |
| mouse α-SMA-F | 5'-GGCACCACTGAACCCTAAGG-3' |
| mouse α-SMA -R | 5'-ACAATACCAGTTGTACGTCCAGA-3' |
| mouse Calponin-F | 5'-GACGGGATCATTCTTTGCGAA-3' |
| mouse Calponin-R | 5'-CCCCATACTTGGTAATGGCTTTG-3' |
| mouse SM22-F | 5'-ACTACCGTGGAGATCCCAA-3' |
| mouse SM22-R | 5'-TTTCTAACTGATGATCTGCC-3' |
| mouse PCNA-F | 5'-TTGCACGTATATGCCGAGACC-3' |
| mouse PCNA-R | 5'-GGTGAACAGGCTCATTCATCTCT-3' |
| mouse FSCN1-F | 5'-GACTGCGAAGGTCGCTACC-3' |
| mouse FSCN1-R | 5'-CTGATCGGTCTCTTCATCCTGA-3' |
| mouse KLF4-F | 5'-ACCAAGAGTTCTCATCTCAAGG-3' |
| mouse KLF4-R | 5'-GTAGTGCCTGGTCAGTTCATC-3' |
| human pri-miR-143-F | 5'-TGGTCCTGGGTGCTCAAATG-3' |
| human pri-miR-143-R | 5'-AGCACTTACCACTTCCAGGCT-3' |
| human pri-miR-145-F | 5'-TCCAGCTGGTCCTTAGGGACA-3' |
| human pri-miR-145-R | 5'-TTGAACCCTCATCCTGTGAGC-3' |
| mouse pri-miR-143 F2 | 5'-ACTCCTCCTGCCCAAGAAGA-3' |
| mouse pri-miR-143 R2 | 5'-TCCCATGCCAACACTTACCA-3' |
| mouse pri-miR-145 F2 | 5'-AGAGAACTGCTGGTCCCTAG-3' |
| mouse pri-miR-145 R2 | 5'-TACTCCCCGGAAGGAAATGC-3' |
| mouse ki67-F | 5'-CAAGGCGAGCCTCAAGAGATA-3' |
| mouse ki67-R | 5'-TGTGCTGTTCTACATGCCCTG-3' |
| rat hnRNPA2B1-F | 5'-ACCAGCAACCTTCTAACTAC-3' |
| rat hnRNPA2B1-R | 5'-CCACTTCCTCCACTTCCT-3' |
| miR-143-3p-F | 5'-CTGGGTGAGATGAAGCACTG-3' |
| miR-143-3p-RT | 5'-GTGCAGGGTCCGAGGTCAGAGCCACCTGGGCAATTTTTTTTTTTGAGCTA-3' |
| miR-143-5p-F | 5'-TTCGGGTGCAGTGCTGCAT-3' |
| miR-143-5p-RT | 5'-GTGCAGGGTCCGAGGTCAGAGCCACCTGGGCAATTTTTTTTTTTACCAGA-3' |
| miR-145-3p-F | 5'-CTGGGGGATTCCTGGAAATAC-3' |
| miR-145-3p-RT | 5'-GTGCAGGGTCCGAGGTCAGAGCCACCTGGGCAATTTTTTTTTTTAGAACA-3' |
| miR-145-5p-F | 5'-CTGGGGTCCAGTTTTCCCAGGA-3' |
| miR-145-5p-RT | 5'-GTGCAGGGTCCGAGGTCAGAGCCACCTGGGCAATTTTTTTTTTTAGGGAT-3' |
| miR-204-5p-F | 5'-CTGGGTTCCCTTTGTCATCCT-3' |
| miR-204-5p-RT | 5'-GTGCAGGGTCCGAGGTCAGAGCCACCTGGGCAATTTTTTTTTTTAGGCAT-3' |
| miR-129-2-3p-F | 5'-CCGGGAAGCCCTTACCCCAAA-3' |
| miR-129-2-3p-RT | 5'-GTGCAGGGTCCGAGGTCAGAGCCACCTGGGCAATTTTTTTTTTTATGCTT-3' |
| miR-149-5p-F | 5'-CTGGGTCTGGCTCCGTGTCTTC-3' |
| miR-149-5p-RT | 5'-GTGCAGGGTCCGAGGTCAGAGCCACCTGGGCAATTTTTTTTTTTGGGAGT-3' |
| miR-28-5p-F | 5'-CTGGGAAGGAGCTCACAGTCT-3' |
| miR-28-5p-RT | 5'-GTGCAGGGTCCGAGGTCAGAGCCACCTGGGCAATTTTTTTTTTTCTCAAT-3' |
| miR-184-F | 5'-CTGGGTGGACGGAGAACTGAT-3' |
| miR-184-RT | 5'-GTGCAGGGTCCGAGGTCAGAGCCACCTGGGCAATTTTTTTTTTTACCCTT-3' |
| miR-425-5p-F | 5'-CTGGGAATGACACGATCACTC-3' |
| miR-425-5p-RT | 5'-GTGCAGGGTCCGAGGTCAGAGCCACCTGGGCAATTTTTTTTTTTCAACGG-3' |
| miR-23a-3p-F | 5'- TCGGATCACATTGCCAGGG-3' |
| miR-23a-3p-RT | 5'-GTGCAGGGTCCGAGGTCAGAGCCACCTGGGCAATTTTTTTTTTTGGAAAT-3' |
| miR-129-5p-F | 5'-CTGGGCTTTTTGCGGTCTGGG-3' |
| miR-129-5p-RT | 5'-GTGCAGGGTCCGAGGTCAGAGCCACCTGGGCAATTTTTTTTTTTGCAAGC-3' |
| miR-328a-3p-F | 5'-CTGGGCTGGCCCTCTCTGCCC-3' |
| miR-328a-3p-RT | 5'-GTGCAGGGTCCGAGGTCAGAGCCACCTGGGCAATTTTTTTTTTTACGGAA-3' |
| SnoRNA202-F | 5'-GTACTTTTGAACCCTTTTCCAT-3' |
| SnoRNA202-RT | 5'-GTGCAGGGTCCGAGGTCAGAGCCACCTGGGCAATTTTTTTTTTTCATCAG-3' |
| Snord44-F | 5'-TGGCCTGGATGATGATAAGCA-3' |
| Snord44-RT | 5'-GTGCAGGGTCCGAGGTCAGAGCCACCTGGGCAATTTTTTTTTTTAGTCAG-3' |
| Universal miRNA primer-R | 5'-CAGTGCAGGGTCCGAGGT-3' |
| Universal Taqman probe | 56-FAM/CAGAGCCAC/ZEN/CTGGGCAATTT/3IABkFQ |

Supplementary Table S3

Primers used for sequencing

| mMETTL3-ORF-F1 | 5'-ATGTCGGACACGTGGAGCTCTA-3' |
| --- | --- |
| mMETTL3-ORF-R1 | 5'-CGCTTAGCTTTGTAAGGAAGTG-3' |

Supplementary Table S4

Primary antibodies used in western blotting

| Name | Brand, Catalog number | Dilution |
| --- | --- | --- |
| METTL3 | Abcam, ab195352 | 1:1000 |
| METTL14 | Proteintech, 26158-1-AP | 1:1500 |
| WTAP | Proteintech, 10200-1-AP | 1:1000 |
| FTO | Abcam, ab92821 | 1:1000 |
| ALKBH5 | Proteintech, 16837-1-AP | 1:1000 |
| Smoothelin | Santa Cruz, SC-376902 | 1:1000 |
| α-SMA | Abcam, ab32575 | 1:1000 |
| Calponin | Cell Signaling Technology, 17819S | 1:1000 |
| SM22 | Abclonal, A6760 | 1:1000 |
| β -actin | Servicebio, GB12001 | 1:2500 |
| MMP2 | Proteintech, 10373-2-AP | 1:1000 |
| PCNA | Proteintech, 10205-2-AP | 1:1000 |
| FSCN1 | Absin, abs115837 | 1:1000 |
| KLF4 | Absin, abs117317 | 1:1000 |
| Horseradish Peroxidase-conjugated AffiniPure Goat Anti-Rabbit IgG | Jackson ImmunoResearch, 170-6515 | 1:10000 |
| Horseradish Peroxidase-conjugated AffiniPure Goat Anti-Mouse IgG | Jackson ImmunoResearch, 115-005-003 | 1:10000 |

Supplementary Table S5

The predicted m^6^A sites of primary miR-143 and miR-145 sequences in human (hsa), rat (rno) and mouse (mmu).

Note: The m^6^A consensus was underlined and the predicted m^6^A was marked in red. The mature miRNA sequences (-5p and -3p) were marked with gray shading, respectively.

| primary hsa-miR-143 (5'-3')  GTCCTCCTTCTAAATTAAGCTGTTCTTGACAAGAAAAGGAAAGAAACAAAGAAAAGAAGAGAAAAAAGGTCAAGGTTTGGTCCTGGGTGCTCAAATGGCAGGCCAC**AGACA**GGAAACACAGTTGTGAGGAATTACAACAGCCTCCCGGCCAGAGCTGGAGAGGTGGAGCCCAGGTCCCCTCTAACACCCCTTCTCCTGGCCAGGTTGGAGTCCCGCCACAGG  CCACCAGAGCGGAGCAGCGCAGCGCCCTGTCTCCCAGCCTGAGGTGCAGTGCTGCATCTCTGGTCAGTTGGGAGTCTGAGATGAAGCACTGTAGCTCAGGAAGAGAGAAGTTGTTCTGCAGCCATCAGCCTGGAAGTGGTAAGTGCTGGGGGGTTGTGGGGGGCCATAACAGGAA**GGACA**GAGTGTTTCCAGACTCCATACTATCAGCCACTTGTGATGCTGGGGAAGTTCCTCTACACAAGTTCCCCTGGTGCCACGATCTGCTTCACGAGTCTGGGCATGTCCTGACTCCTCTGT |
| --- |
| Primary hsa-miR-145 (5'-3')  GGAATACACATGAGCCGTGCAGACAGCAGAGGGCAGTCCTGGGGGTGGGGGCGCCAGAGGGTTTCCGGTACTTTTCAGGGCAATTGAAGTTCCGGTCACTACACCCCCCCAGAGCAATAAGCCACATCCGGCGACGTGTGGCACCCCACCCTGGCTGCTACAGATGGGGCTGGATGCAGAAGA**GAACT**CCAGCTGGTCCTTAG**GGACA**CGGCGGCCTTGGCGCTGAAGGCCACTCGCTCCCACCTTGTCCTCACGGTCCAGTTTTCCCAGGAATCCCTTAGATGCTAAGATGGGGATTCCTGGAAATACTGTTCTTGAGGTCATGGTTTCACAGCTGGATTTGCCTCCTTCCCACCCCACAGTTGCCCCCCAATGGGGCCTCGGCTGGCTCACAGGATGAGGGTTCAAGAAGAAGGCTGTCCCTGGAGGTAAGAGGGCTTATGAACCATGTTCCAAACCTTTGCGTTGCTTTTCTTTCCATCGTGTCTATTTCATAACATCCCTGTGAGGCTG |
| Primary rno-miR-143 (5'-3')  AACTGCACACTTCAGATGGGTAGGGTCCATCTCAAGAAAGC**TAACT**TTAAA**AGACA**AA**GGACA**CGAAGATGGACGTTTCCTTCCCAGAGGTCTCCAGGGCGTGTCCAGACCAGTATAGGAGGCAGACCACTGCGCATGCTCATTCTCCTGCCCAGGAAGAAAGCCGTGGCCTGAGCGCGGAGCGCCTGTCTCCCAGCCTGAGGTGCAGTGCTGCATCTCTGGTCAGTTGGGAGTCTGAGATGAAGCACTGTAGCTCAGGAAGGGAGAAGATGTTCTGCAGCCGTCGCCG**GGACA**TGGTAAGTGTTGGTGAGGGGTAGCGGGCCCGATCGGTTGTCCCTAGAGTCCGCTGCAGCAACTTGTGGTGATGTTCCTTGACTCAGGCTGTACCAGGGCTGAGCACTCTTATGCCATCCGTAACAGTAAACGCTGGTTCATTCAATGGCCGCCAGCACCAACACACTGCCTTTACTTCCTCAGCCCAGAGA**AGACA**CTGCTAACAAACAAACAAACAAACAAACCGCGGC |
| Primary rno-miR-145 (5'-3')  AGGGA**GGACT**GGTCCCAAATGCTTCCTGACCTCAGGGGAGGTACTTTCCAAGCCACTCAGAGCTCTGGTTGCTCTCCCCAGAGTAGAGAGCTGCATCCTGAGAAGTGAGGTGCATATAGCACCCCACACTGGCTGCCAGAGCTGGCGCCGGATGCAGGGAAAACTGCTCGTCCCTAGGGGTGGGCGTGGCACTGCTGAAGGCATCTCTCTCCCACCTTGTCCTCACGGTCCAGTTTTCCCAGGAATCCCTTGGATGCTAAGATGGGGATTCCTGGAAATACTGTTCTTGAGGTCATGGCTTAACAGCTGGATCTGCCTCCTTCCCACCCTGACATTTCCTCCCAGGGAGAAGGGAGGAGGAGGAGGTGCATCGGAGGGAGGTAAGCGGTCTTGGGACCTCTTCTGACCTTTGTGTTGCTTTTTTCCCACTGCGTCCATATTTCCTAACATCCCGGGGAGGTTGGATGTGGGAACCTCA**GAACT**GCTTCACTCTTGAGAAACTTGTCTCGAGTCACAGTGGTTGAACC |
| Primary mmu-miR-143 (5'-3')  GCAGGCAGGCCCTGGGTTCCATCTTTAGCACTGAAAAAAAAAAAATTAACTGCATGCTTGAAATGGGTGGGTCTATCACAAGAAAGCTAACTTAAAACGACAAAA**GGACA**AGAAAGTGGAAATTTCAGTCCCAGAGGGCTCCAGGGTAAGCCAAGACCCGGATAGGAGGCAGACCACTGCGCATGCTCACACTCCTCCTGCCCAAGAAGAAAGCCGCGCGGCCTGCGTGCGGAGCGCCTGTCTCCCAGCCTGAGGTGCAGTGCTGCATCTCTGGTCAGTTGGGAGTCTGAGATGAAGCACTGTAGCTCAGGAAGGGAGAAGATGTCCTGCAGCCGTCACCGGGACGTGGTAAGTGTTGGCATGGGATAGC**GGACT**AGATAGGTTGTCCCTAGATTCTGCTCCAGCAGCTTGTGGCGATGTTCCTGGGCTCAAGCTGTACCAGGGCCGATCACTCTTGTGCCCTCTACAACAGTAAGCTCTGGTCCATTCAGGGGCTGCCAGCACCAACACCCTGCCTCTA |
| Primary mmu-miR-145 (5'-3')  CTTCCTGACCTTAGGGAAGGTACTTTCCAAGCCACTCAAAGCTCTAGTTGCTCTTCCCAGAGCAGGACGCTGCATCCCGGGAAGGAGGGTGTATACAGCGCCCCACGGTGGATGCCGAGA**GAACT**GCTGGTCCCTAGGGGCGTGGCACGTGCTGAAGGCATCTCTCTCTCTCCCACCTTGTCCTCACGGTCCAGTTTTCCCAGGAATCCCTTGGATGCTAAGATGGGGATTCCTGGAAATACTGTTCTTGAGGTCATGGCTTAGCAGCTGGATCTGTCTCCCTCCCACCCTGGCATTTCCTTCCGGGGAGTAGGGAGGAGGAGGCGGTTCGTCGGAGGTAAGCGGTCTTGGGAGCTCTCTTCTGACCTTTGTATAGCTTTTCTTTCCGCCCTGTCCATATTTCCTAACATCCCCAGGAGGGAGGCTGGATGTGGGAAGCTCA**GAACT**GCCTCACTCTTGAG**AAACT**CGTCTCAAGACACAGTGGTTGAAAACC**AGACT**ATGAGACGGCTCCCCTCCACTC |

Supplementary Figure S1


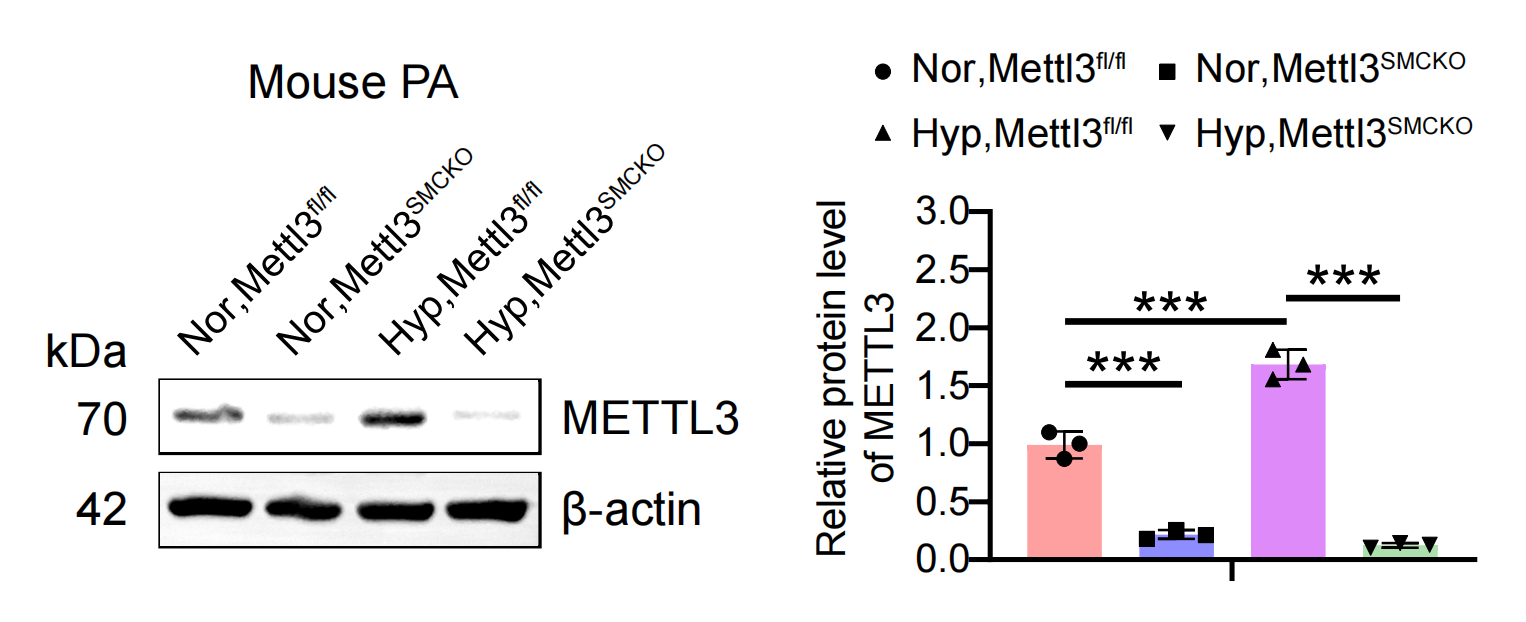


**Fig. S1** The protein levels of METTL3 in mouse PAs were evaluated by western blotting. β-actin was used as a loading control for western blotting (n=3). Data were analyzed by using a one-way ANOVA followed by Tukey's multiple comparisons test. Statistical significance is denoted by *** *P* < 0.001.

Supplementary Figure S2


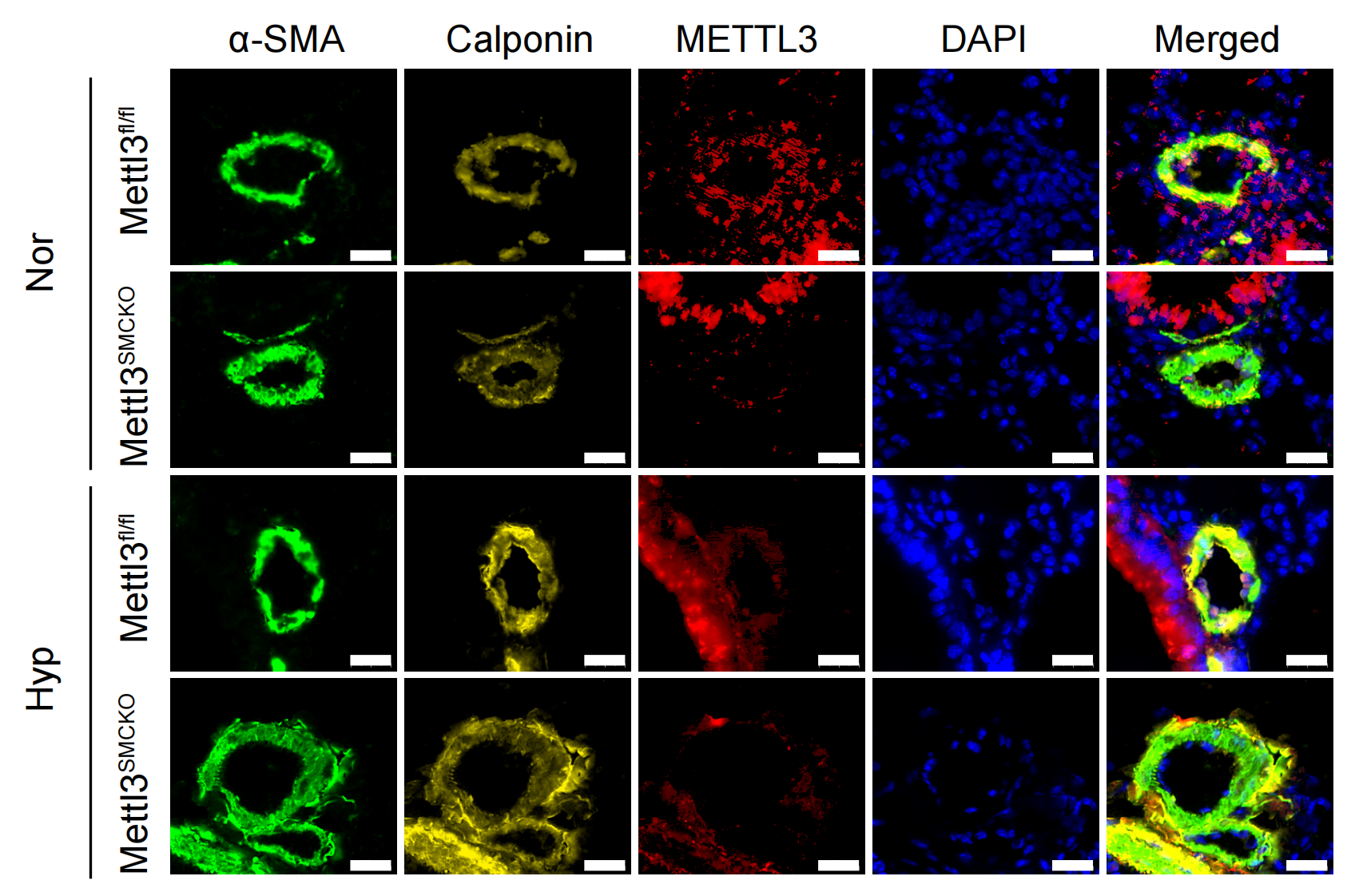


**Fig. S2** Immunostaining analysis of *Mettl3* knockout mice. Representative immunostaining with antibodies against METTL3, α-SMA and Calponin in the pulmonary arteries (PAs) of *SMMHC-Cre^ERT2^;Mettl3^fl/fl^ (Mettl3^SMCKO^)* and the control mice *Mettl3^fl/fl^* under both normoxia and hypoxia. Scale bars, 20 μm.

Supplementary Figure S3


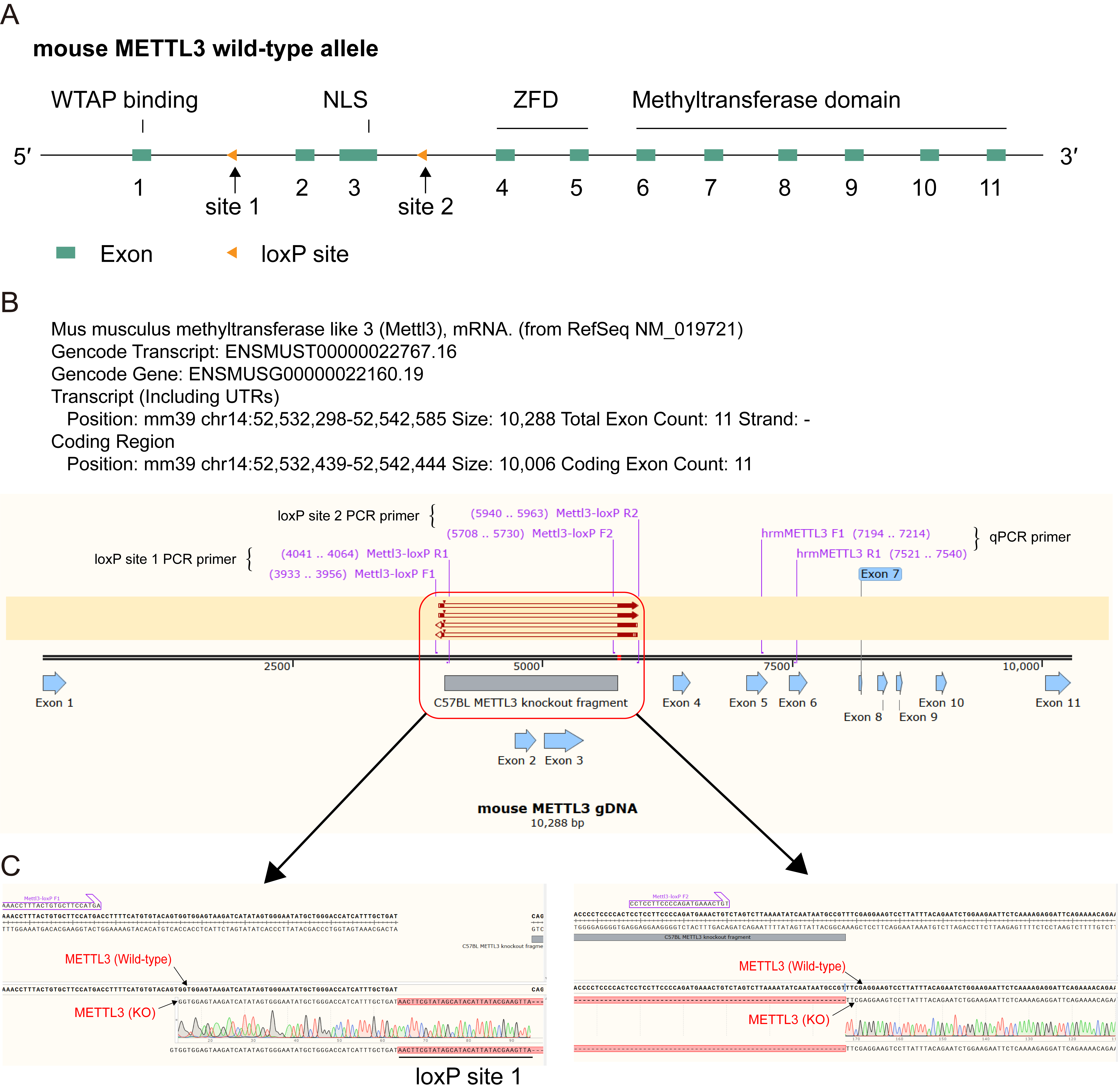


**Fig. S3** *Mettl3^SMCKO^* mouse genotype. **A** Schematic of *Mettl3^fl/fl^* mouse genotype. Two loxP sites around exon 2/3 were shown. The WTAP-binding domain, nuclear localization signal (NLS), zinc finger domain (ZFD) and methyltransferase domain were depicted. **B** The knockout of *mettl3* in *Mettl3^SMCKO^* mouse genomic DNA (gDNA) was assessed by PCR and DNA sequencing using primer set of Mettl3-loxP F1 and Mettl3-loxP R1. **C** The sequence alignment analysis of *Mettl3* in *Mettl3^SMCKO^* mouse gDNA. The first line of the DNA sequence represents the wild-type METTL3 sequence, and the second line represents the knockout version of METTL3. LoxP site 1 was retained after the exon 2/3 fragment of *Mettl3* was removed. The PCR and sequencing primers used are listed in Supplementary Table S1.

Supplementary Figure S4

**
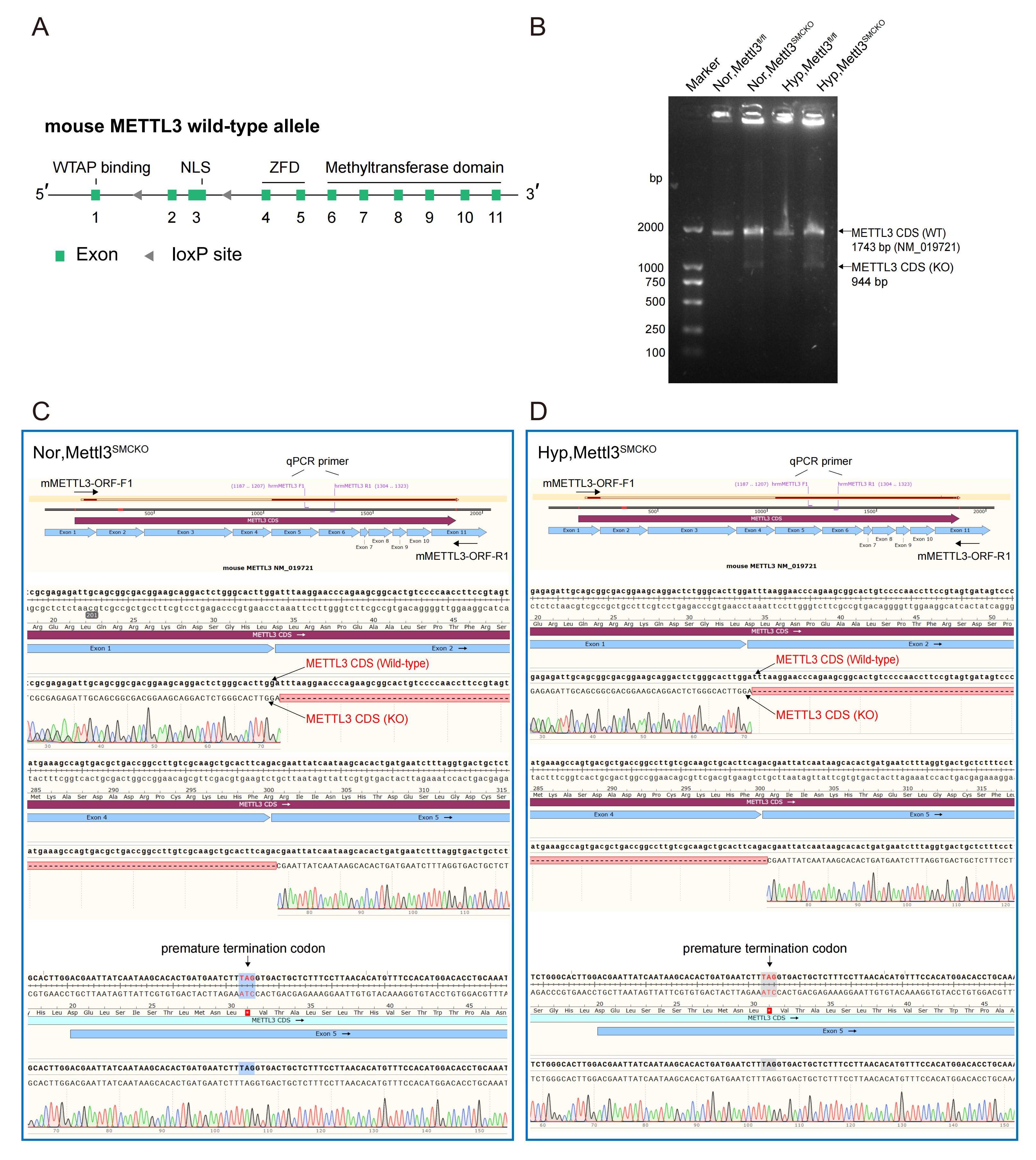
**

**Fig. S4** Sequence analysis of *Mettl3* knockout in mRNA level. **A** Schematic of *Mettl3* conditional knockout by using Cre-*loxP* system. The WTAP-binding domain, nuclear localization signal (NLS), zinc finger domain (ZFD) and methyltransferase domain were depicted. **B** The coding sequence (CDS) of *Mettl3* was amplified using RT-PCR from the lung tissues of both *Mettl3^SMCKO^* and *Mettl3^fl/fl^* mice, followed by analysis with agarose gel electrophoresis. **C, D** Sequencing analysis of the *Mettl3* CDS was performed for normoxic *Mettl3^SMCKO^* (**C**) and hypoxic *Mettl3^SMCKO^* (**D**). In addition to exon 2 and 3, exon 4 was also deleted in *Mettl3* CDS, likely due to alternative splicing. This leads to the production of a premature termination codon at exon 5. WT: wild-type. The sequencing primers used are listed in Supplementary Table S3.

Supplementary Figure S5


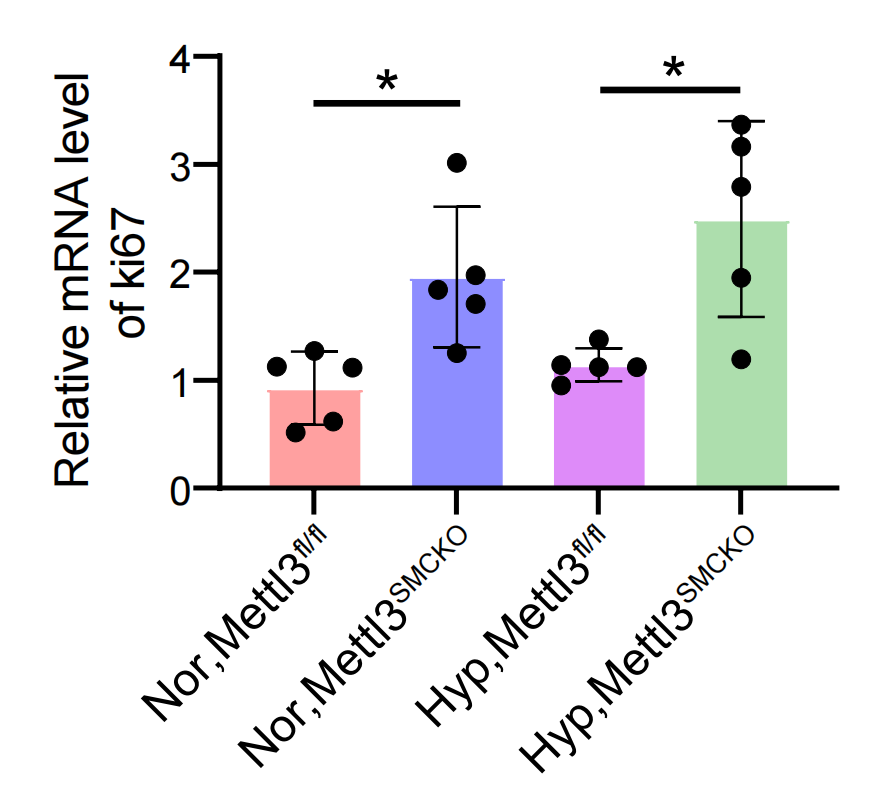


**Fig. S5** The mRNA levels of ki67 in mouse PAs were evaluated by qRT-PCR (n=5). β-actin was used as an internal reference for qRT-PCR. Nor: normoxia; Hyp: hypoxia. Data were analyzed by using a one-way ANOVA followed by Tukey's multiple comparisons test. Statistical significance is denoted by * *P* < 0.05.

Supplementary Figure S6


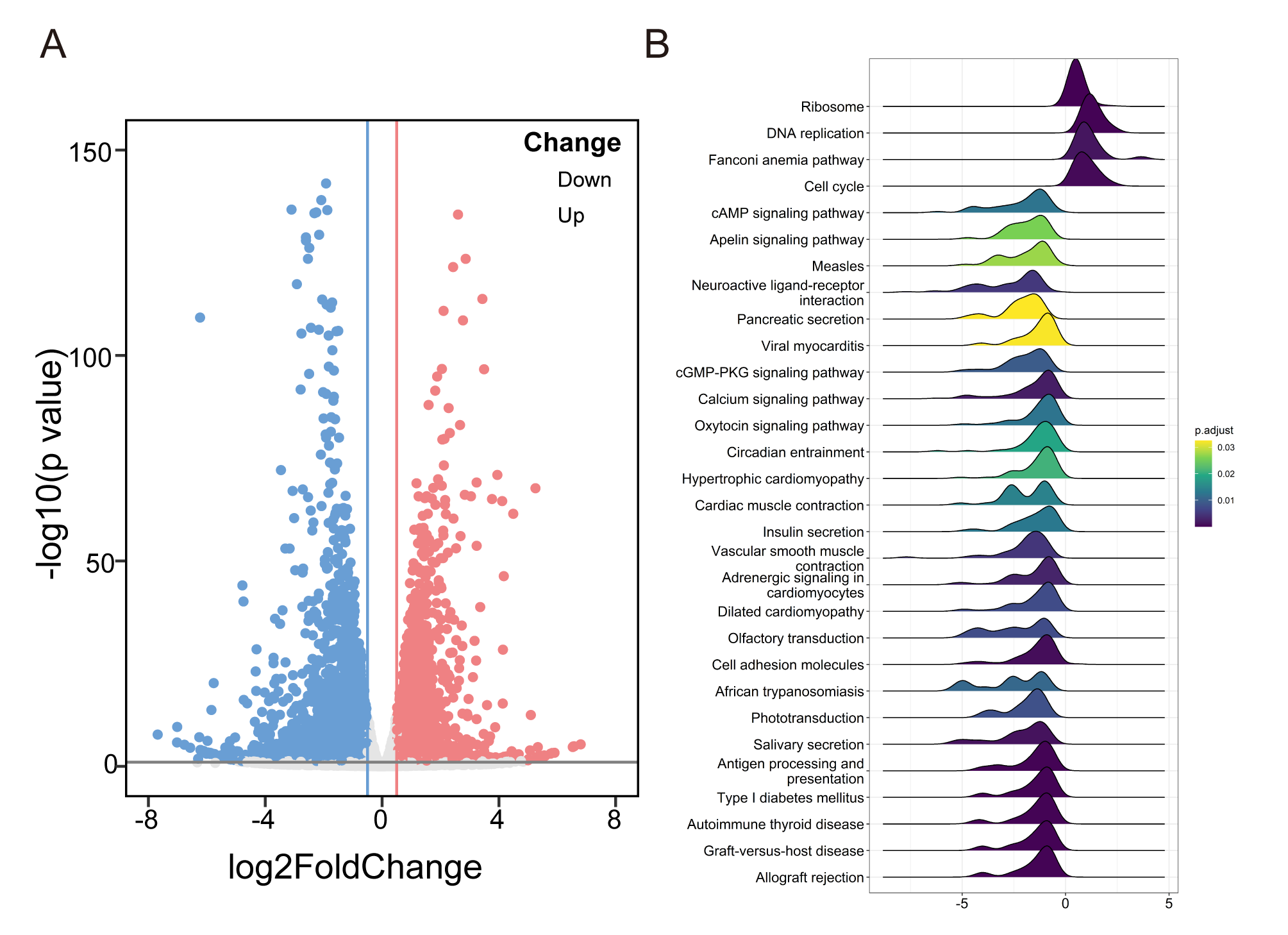


**Fig. S6** Transcriptome analysis of METTL3-silenced rPASMCs. **A** Volcano plot of differentially expressed genes (DEGs) in rPASMCs infected with either shMETTL3 or shNC lentiviruses. The horizontal axis represents mean log2FoldChange; the vertical axis represents -log10(p-value) of DEseq2 of shMETTL3 samples relative to shNC samples; Red color dots stand for upregulation genes. Blue color dots stand for downregulation genes. **B** KEGG analysis of the DEGs in the same rPASMCs samples.

Supplementary Figure S7


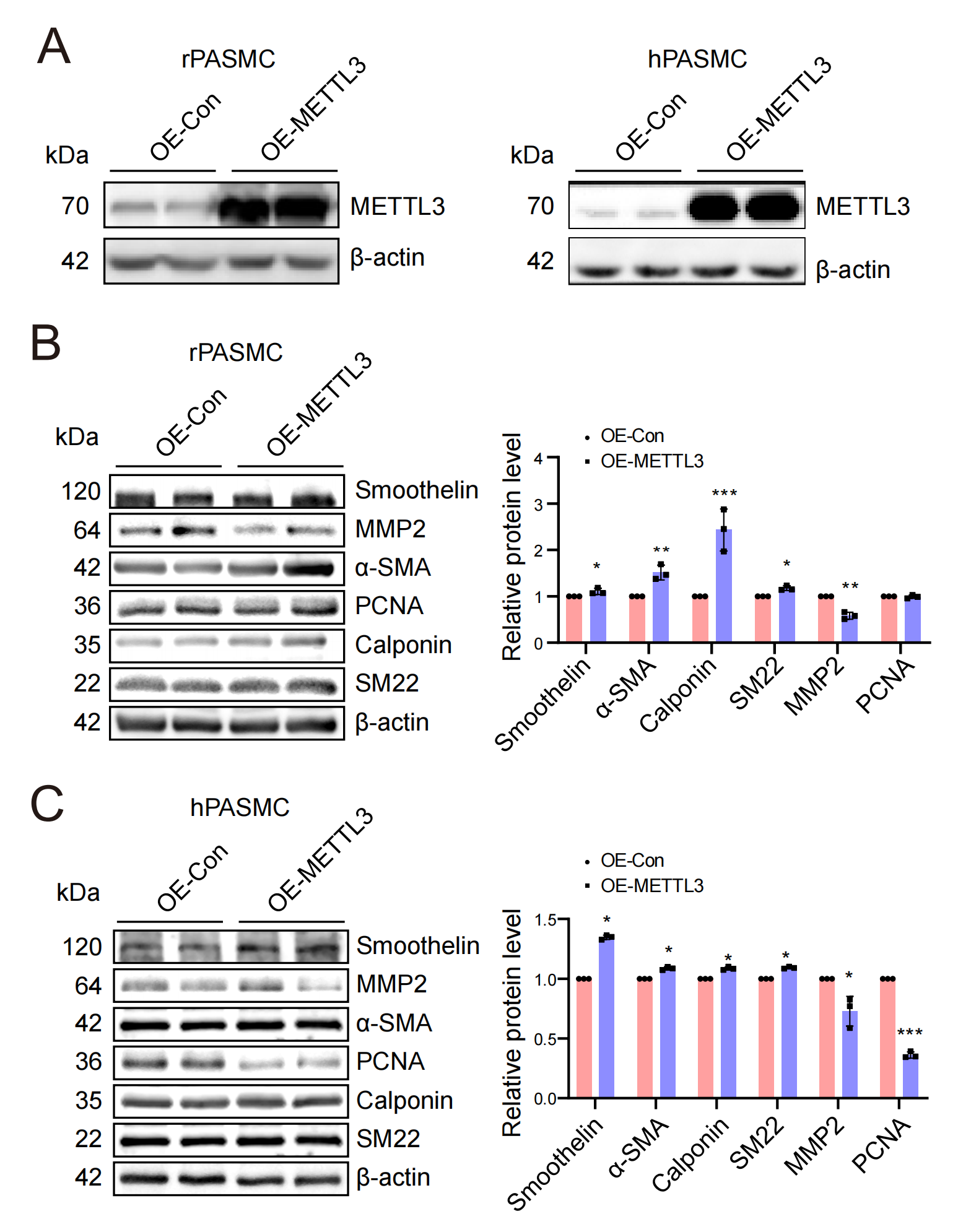


**Fig. S7** The effects of overexpression of METTL3 on PASMC functions. **A** The overexpression of METTL3 in rPASMCs (**A**, left) and hPASMCs (**A**, right) was verified by western blotting. **B**-**C** The protein levels of α-SMA, SM22, Smoothelin, Calponin, MMP2 and PCNA in rPASMCs (**B**) and hPASMCs (**C**) overexpressing OE-METTL3 compared to the control group (OE-Con) (n=3). β-actin was used as a loading control for western blotting. OE-METTL3: overexpressing METTL3 coding sequence; OE-Con: negative control group. A two-tailed unpaired t test was used to estimate the significance. Statistical significance is denoted by * *P* < 0.05, ** *P* < 0.01 and *** *P* < 0.001.

Supplementary Figure S8


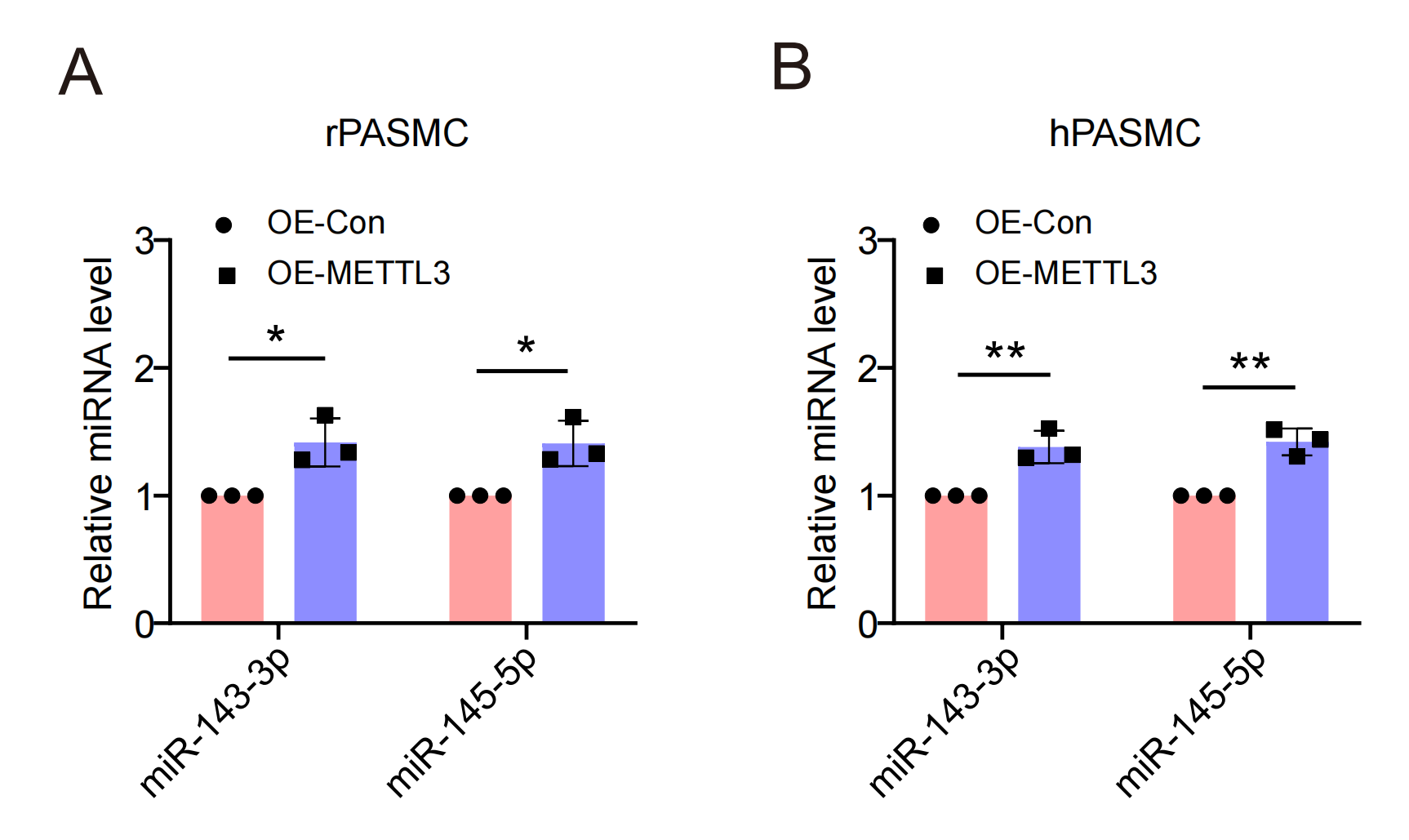


**Fig. S8** The regulatory impact of METTL3 on miR-143/145 expression. **A**-**B** The miR-143-3p and miR-145-5p levels were detected in OE-Con and OE-METTL3 rPASMCs (**A**) and hPASMCs (**B**) by qRT-PCR (n=3). SnoRNA202 and Snord44 were used as internal references in qRT-PCR in rPASMCs and hPASMCs, respectively. Data were analyzed by a two-tailed unpaired t test. Statistical significance is denoted by * *P* < 0.05 and ** *P* < 0.01.

Supplementary Figure S9


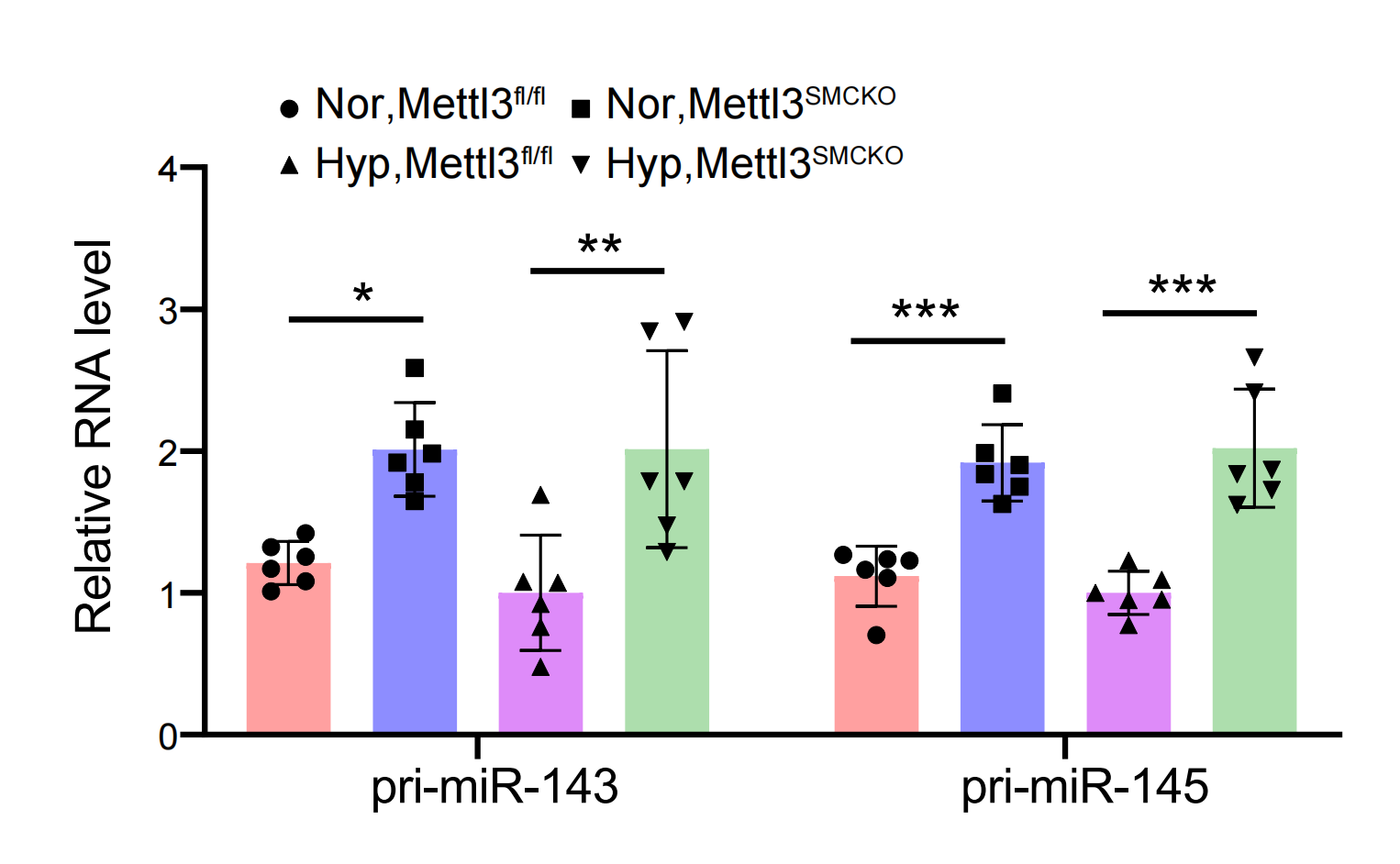


**Fig. S9** The expression of pri-miR-143 and pri-miR-145 in hypoxic PH model. The levels of pri-miR-143 and pri-miR-145 in mouse pulmonary arteries (PAs) were evaluated by qRT-PCR (n=6). β-actin was used as an internal reference for qRT-PCR. Data were analyzed by using a one-way ANOVA followed by Tukey's multiple comparisons test. Statistical significance is denoted by * *P* < 0.05, ** *P* < 0.01 and *** *P* < 0.001.

Supplementary Figure S10

**
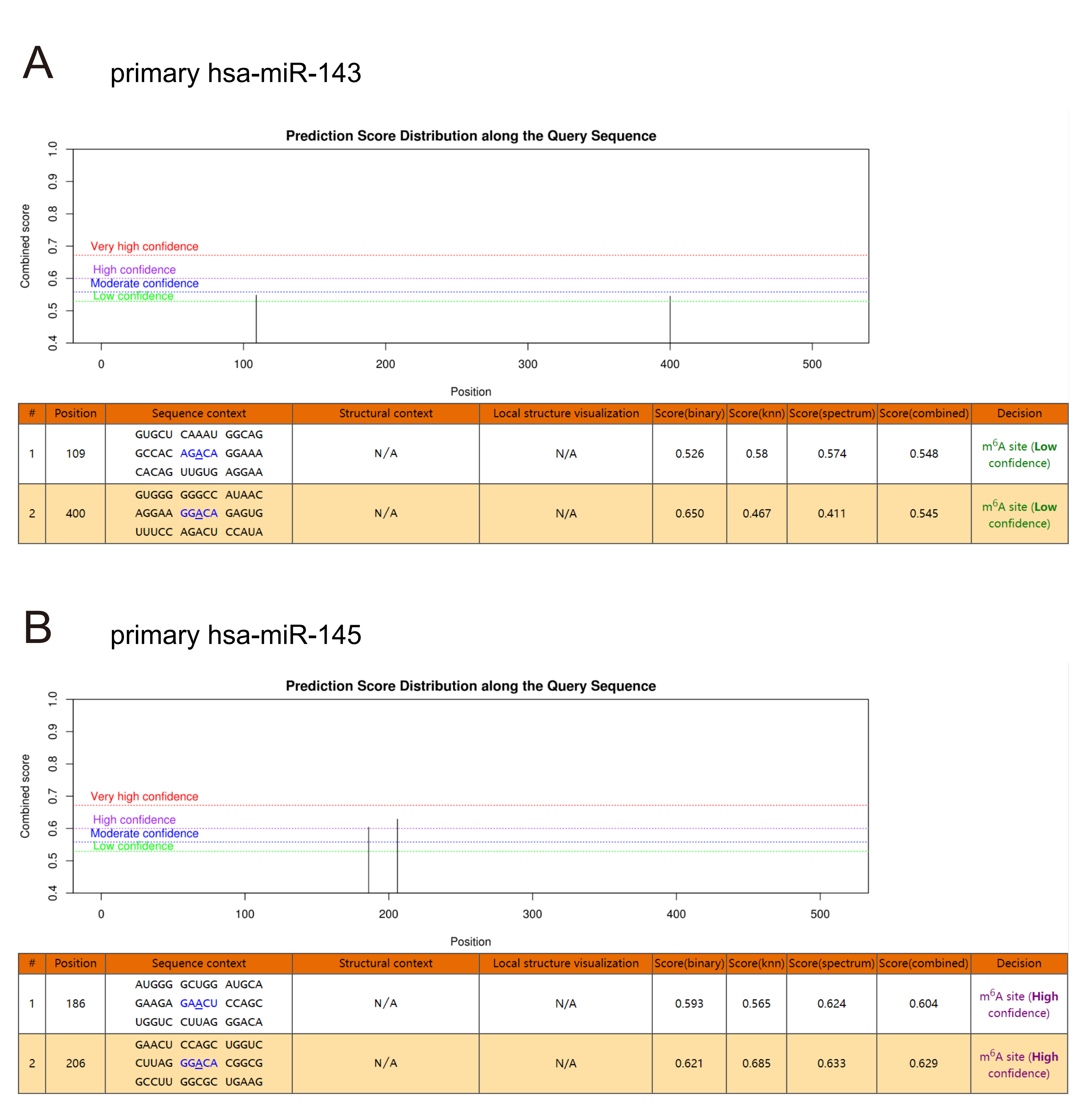
**

**Fig. S10** Prediction of m^6^A sites on human pri-miRNA was performed using SRAMP (<http://www.cuilab.cn/sramp/>). **A** primary hsa-miR-143. **B** primary hsa-miR-145.

Supplementary Figure S11


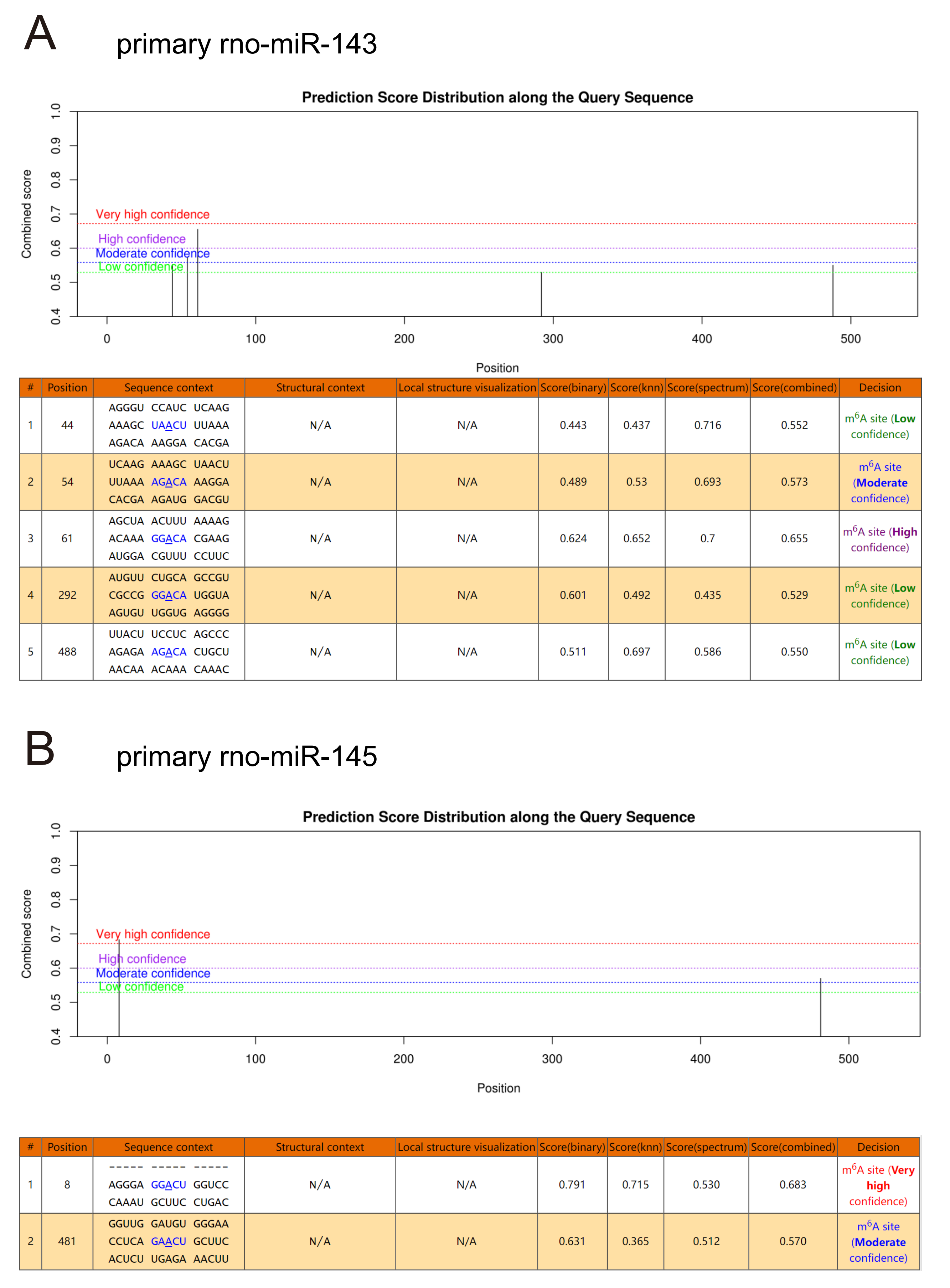


**Fig.** **S11** Prediction of m^6^A sites on rat pri-miRNA was performed using SRAMP (<http://www.cuilab.cn/sramp/>). **A** primary rno-miR-143. **B** primary rno-miR-145.

Supplementary Figure S12


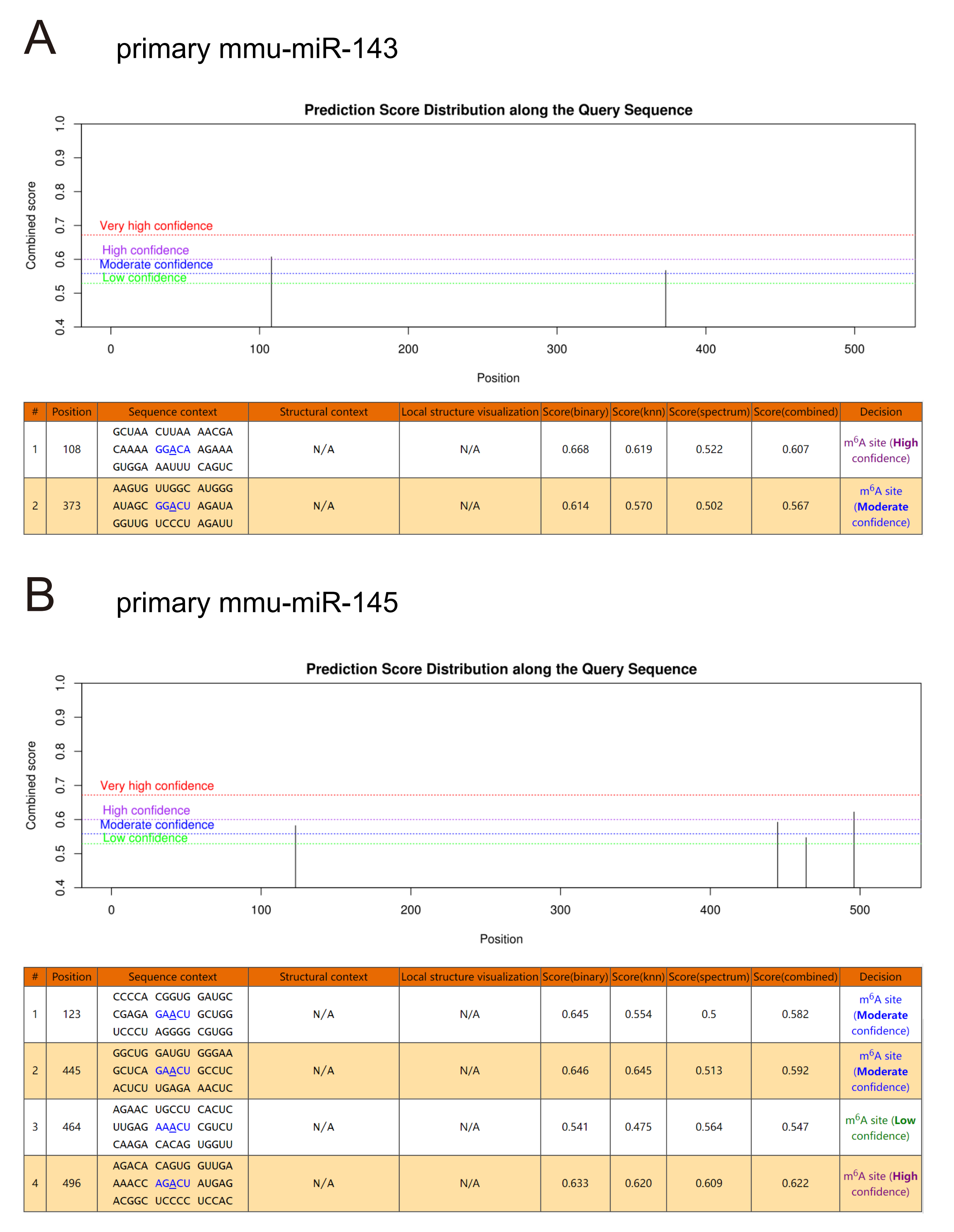


**Fig. S12** Prediction of m^6^A sites on mouse pri-miRNA was performed using SRAMP (<http://www.cuilab.cn/sramp/>). **A** primary mmu-miR-143. **B** primary mmu-miR-145.

Supplementary Figure S13

**
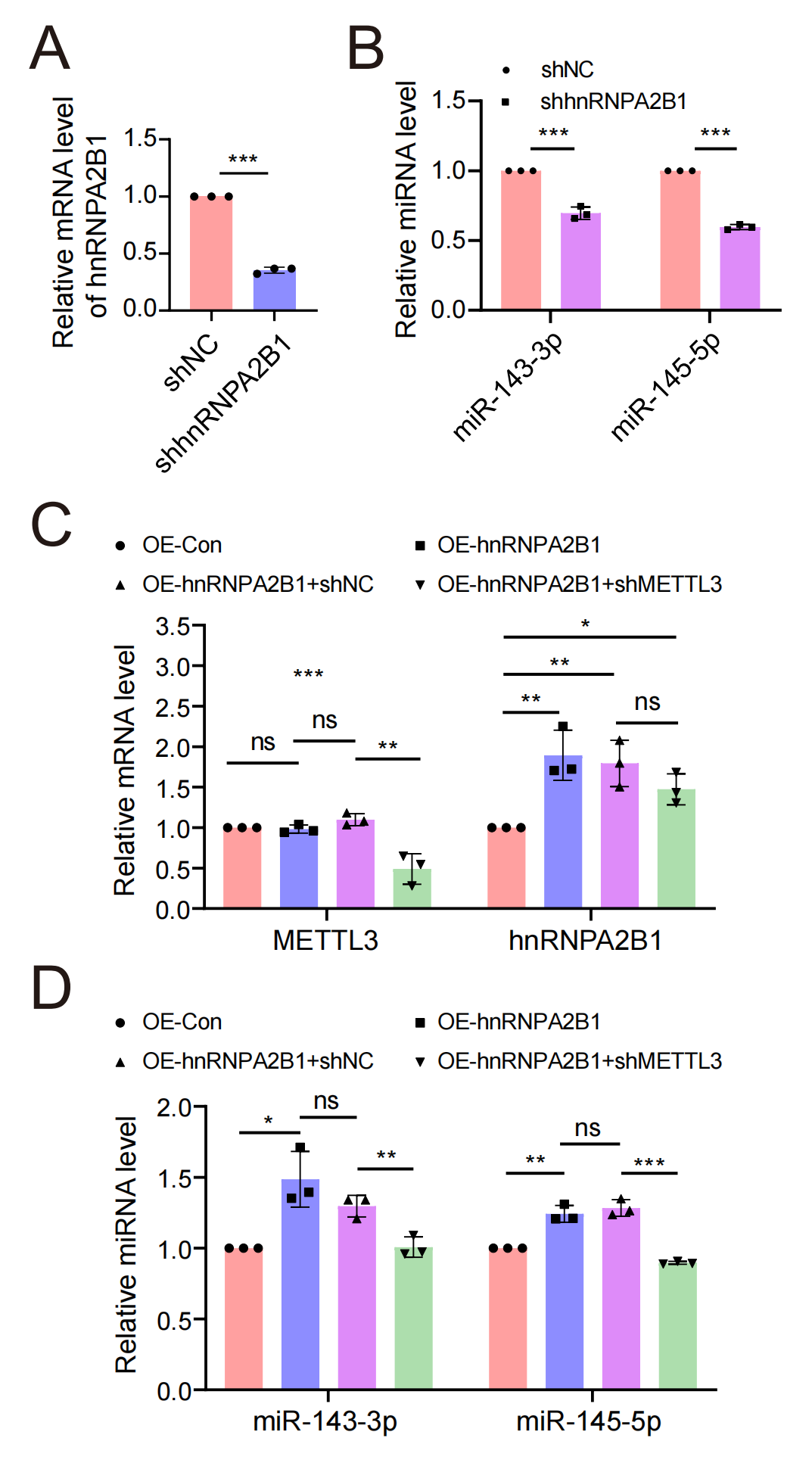
**

**Fig. S13** The effects of hnRNPA2B1 on miR-143/145 expression. **A** hnRNPA2B1 inhibition by shRNA was confirmed with qRT-PCR (n=3). **B** In hnRNPA2B1-silenced rPASMCs, the miR-143-3p and miR-145-5p expression were assayed by qRT-PCR (n=3). **C-D** The expression levels of METTL3, hnRNPA2B1 (**C**), miR-143-3p and miR-145-5p (**D**) were detected by qRT-PCR in OE-Con or OE-hnRNPA2B1 rPASMCs infected with shNC or shMETTL3 lentivirused (n=3). β-actin or snoRNA202 was used as an internal reference in qRT-PCR for mRNA (**A, C**) or miRNA (**B, D**), respectively. A two-tailed unpaired t test was used to estimate the significance. Statistical significance is denoted by * *P* < 0.05, ** *P* < 0.01 and *** *P* < 0.001.

Supplementary Figure S14

**
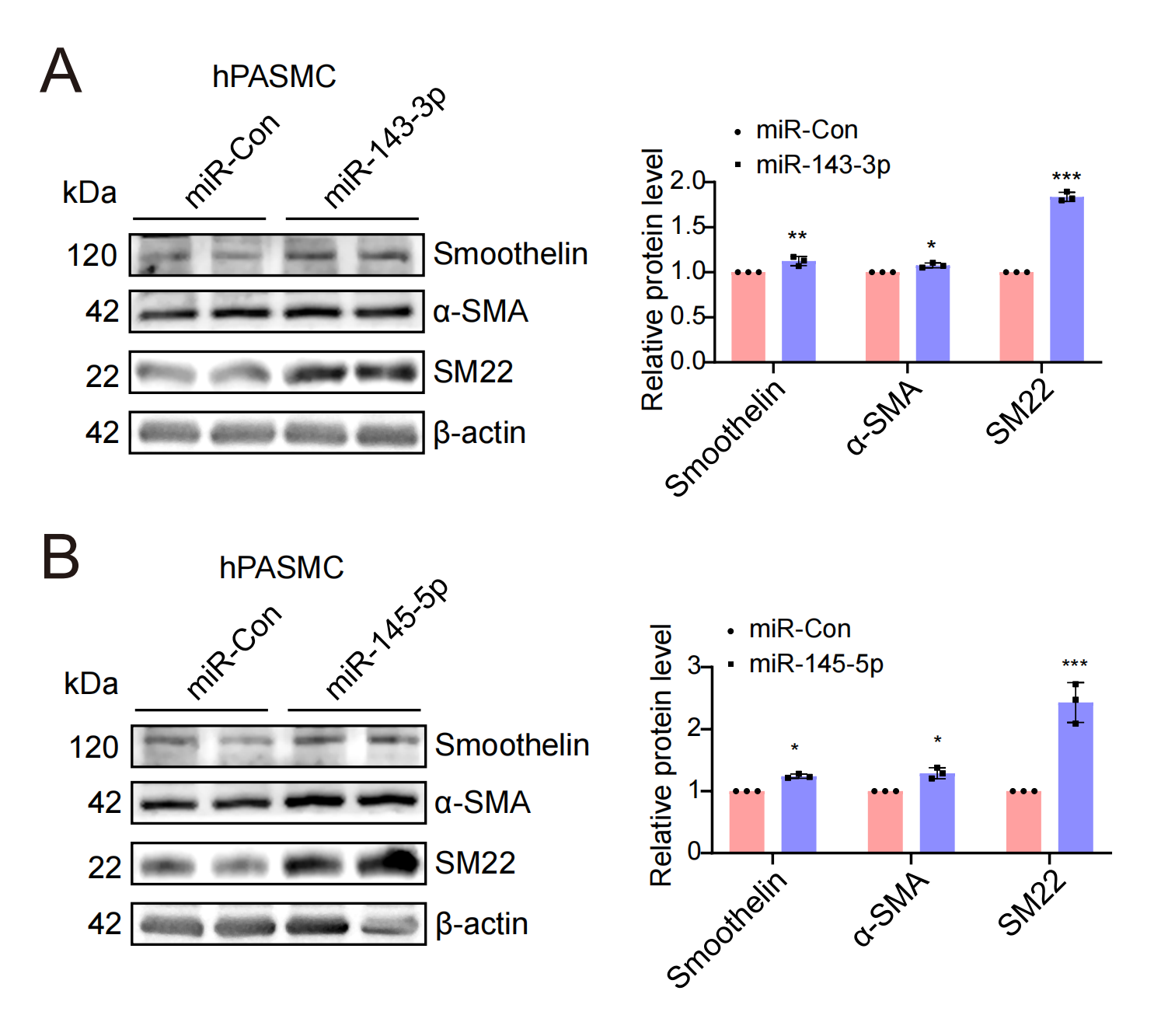
**

**Fig. S14** The effects of miR-143/145 on hPASMCs contractile proteins. **A**-**B** Western blotting analysis was performed to determine the expression levels of SM22, α-SMA, and Smoothelin in hPASMCs transfected with miR-143-3p mimic (**A**) and miR-145-5p mimic (**B**) (n=3). Bar charts represent the relative protein levels. β-actin was used as a loading control for western blotting. Data were analyzed by a two-tailed unpaired t test. Statistical significance is denoted by * *P* < 0.05, ** *P* < 0.01 and *** *P* < 0.001.

Supplementary Figure S15

**
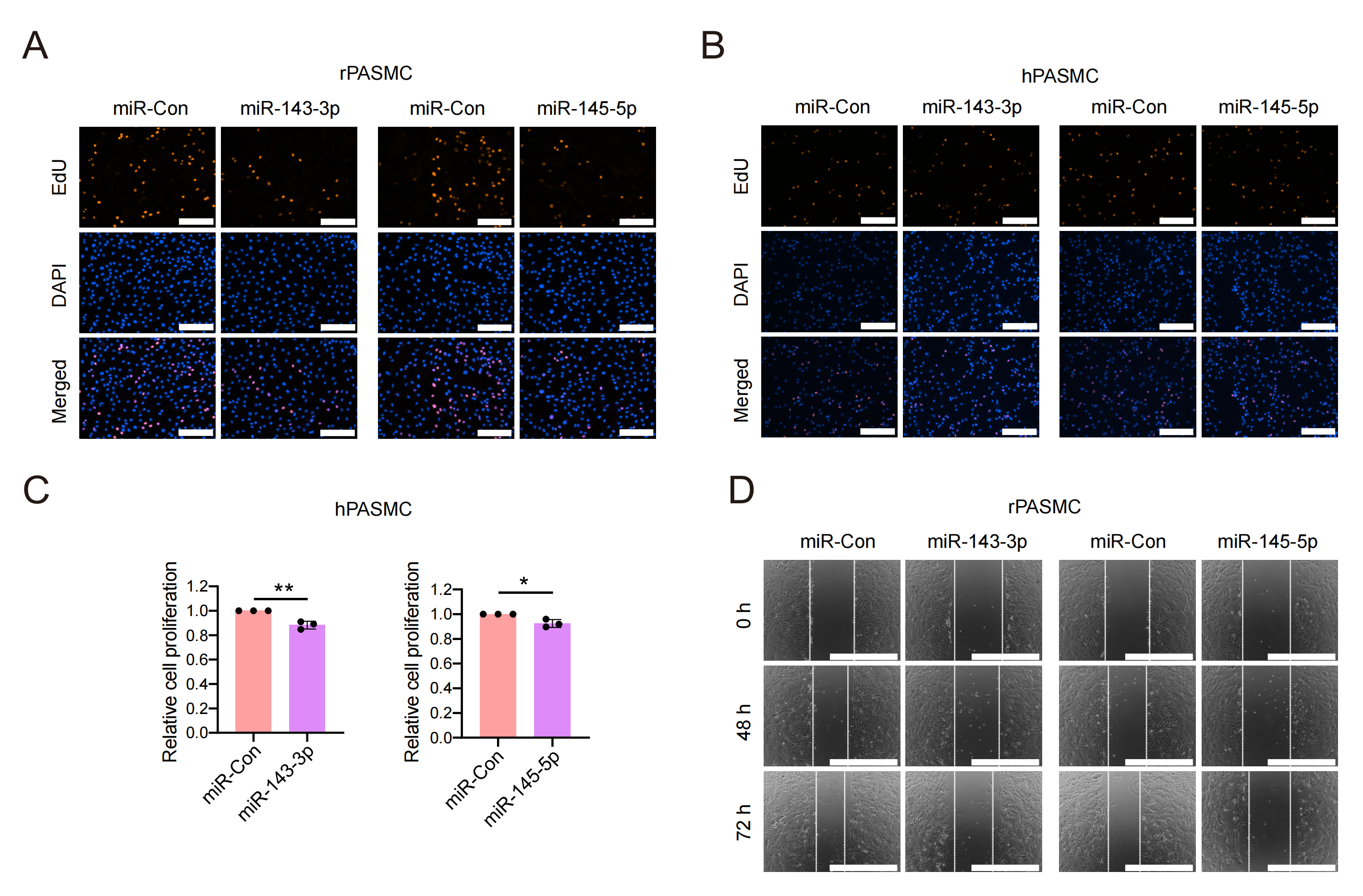
**

**Fig. S15** The effects of miR-143/145 on proliferation and migration in PASMCs. **A**-**C** Representative images of EdU labeling depicts the proliferation of rPASMCs (**A**) and hPASMCs (**B**) upon transfection of miR-143-3p and miR-145-5p mimics. EdU-positive cells was quantified across 10 random fields, with DAPI staining highlighting all cells (n=3). Scale bar represents 200 μm. The bar chart illustrates the proportion of EdU-positive cells in hPASMCs (**C**). **D** Representative images from wound healing assay display the migration of rPASMCs following transfection of miR-143-3p and miR-145-5p mimics (n=3). Scale bar represents 1000 μm. Data were analyzed by a two-tailed unpaired t test. Statistical significance is denoted by * *P* < 0.05 and ** *P* < 0.01.

Supplementary Figure S16

**
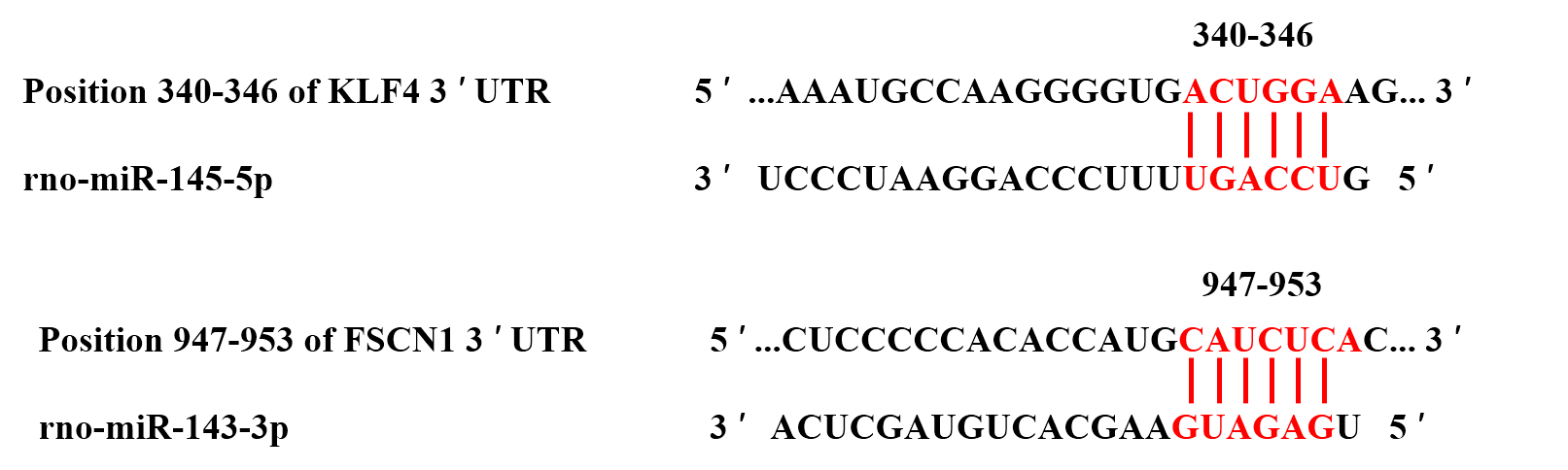
**

**Fig. S16** Schematic of miR-143/145 and targets. The miR-145-5p binding site on the 3 ′ -UTR of KLF4 and the miR-143-3p binding site on the 3 ′ -UTR of FSCN1 were analyzed using TargetScan.

Supplementary Figure S17


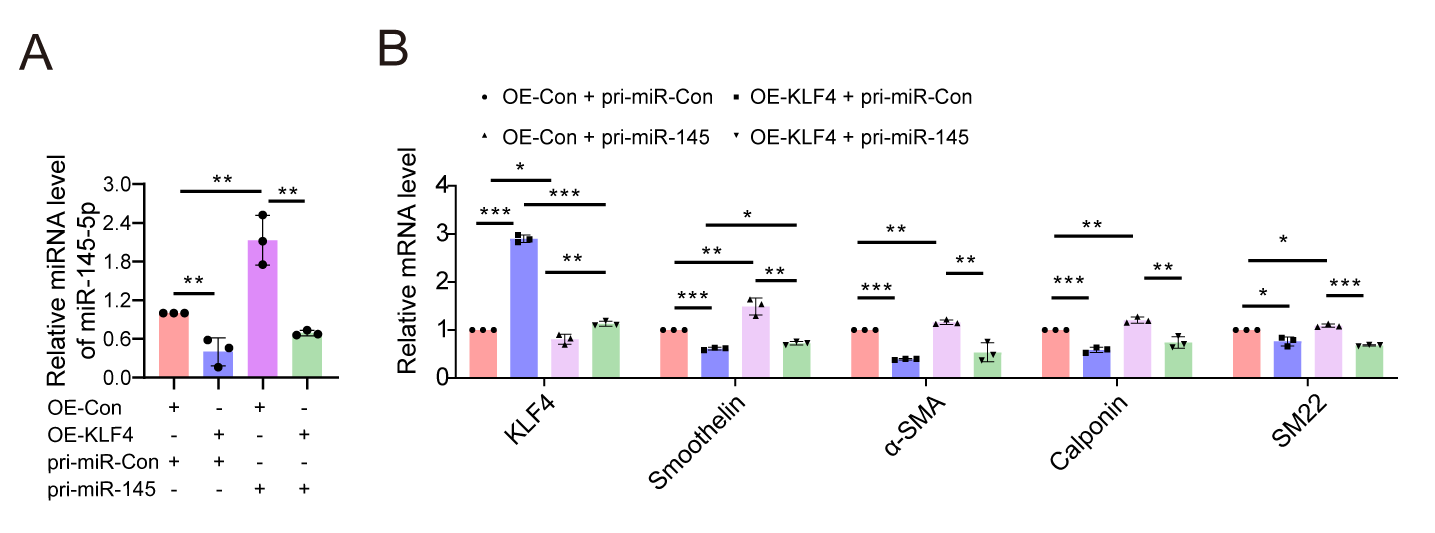


**Fig. S17** overexpression of miR-145-5p reverses the decline in contractile genes induced by KLF4. **A**-**B** The levels of miR-145-5p (**A**), relative mRNA levels of KLF4, Smoothelin, SM22, α-SMA and Calponin (**B**) were detected by qRT-PCR in OE-Con or OE-KLF4 rPASMCs transfected with either a pri-miR-145 plasmid or a control plasmid lacking pri-miRNA (pri-miR-Con) (n=3). snoRNA202 or β-actin was used as an internal reference in qRT-PCR for miRNA or mRNA, respectively. Data were analyzed by using a one-way ANOVA followed by Tukey's multiple comparisons test. Statistical significance is denoted by * *P* < 0.05, ** *P* < 0.01 and *** *P* < 0.001.
